# Validation of the German Five-Factor Narcissism Inventory and Construction of a Brief Form Using Ant Colony Optimization

**DOI:** 10.1177/10731911221075761

**Published:** 2022-02-18

**Authors:** Emanuel Jauk, Gabriel Olaru, Eva Schürch, Mitja D. Back, Carolyn C. Morf

**Affiliations:** 1 Technische Universität Dresden, Germany; 2 Medical University of Graz, Austria; 3 Tilburg University, The Netherlands; 4 University of Bern, Switzerland; 5 University of Münster, Germany

**Keywords:** narcissism, Five-Factor Narcissism Inventory (FFNI), grandiose narcissism, vulnerable narcissism, agentic narcissism, antagonistic narcissism, neurotic narcissism

## Abstract

Narcissism is a multifaceted construct commonly conceptualized as comprising grandiose and vulnerable aspects in a two-factor model. While the manifold correlates of these aspects imposed a challenge for research on the structure of narcissism, recent models converge in a three-factor structure of agentic-extraverted, antagonistic, and neurotic aspects, capturing variance in different conceptualizations and correlates of narcissism. We construct and validate a German adaptation of the Five-Factor Narcissism Inventory (FFNI), a measure assessing these aspects based on the Five-Factor Model. In eight samples (*N* = 2,921), we found the German FFNI to align with both, two- and three-factor models. The factors display good criterion validity with other narcissism measures, (non-)clinical personality dimensions, interpersonal styles, and (mal-)adaptive adjustment. Neurotic and antagonistic narcissism discriminated between individuals with/without mental disorder diagnoses, and displayed a characteristic profile in incarcerated offenders. Since the FFNI is comprehensive but long, we constructed a 30-item brief form (FFNI-BF) optimizing the internal structure and external validity using ant colony optimization. The FFNI-BF displayed good psychometric characteristics and similar, in certain aspects even advantageous criterion validity. We conclude that the German FFNI validly measures key aspects of narcissism, and the FFNI-BF captures these in a concise manner.

## Introduction

Narcissism has fascinated humankind for a long time, putatively because of its manifold consequences for our individual and social lives. In the ancient myth, Narcissus rejects the love of Echo, and is therefore condemned to fall in love with his mirror image, which ultimately leads him to a tragic death. This myth can be seen to reflect two main themes which are evident across a wide range of psychological literature on narcissism, namely grandiosity and vulnerability (Pincus & Lukowitsky, 2010). The grandiose aspects of narcissism are related to egotistical but largely—though by no means exclusively—adaptive functioning in the general population, whereas the vulnerable aspects go along with a variety of intrapersonal and interpersonal problems (Kaufman et al., 2020; Miller et al., 2011). Still, viewed from an interindividual differences perspective, the broad themes of grandiose and vulnerable narcissism, translated into personality traits, remain fuzzy to some extent, as each of them contains a mixture of different aspects which have different relations to relevant aspects of experience and behavior (e.g., Back et al., 2013). Calls have been made for a more fine-grained structure of narcissism (e.g., Ackerman et al., 2011, 2019), and the Five-Factor Narcissism Inventory (FFNI; Glover et al., 2012) has been constructed as an individual differences measure from a Five-Factor Model (FFM) perspective. Here, we present a German translation and validation of the FFNI, providing evidence for its factor structure and extended nomological network (Study 1), and a brief form optimizing its internal structure and validity using ant colony optimization (ACO; Study 2).

### Measuring Individual Differences in Narcissism: From Single Scores to Psychological Profiles

#### Grandiose Narcissism

When thinking of narcissism, most people think of exaggerated feelings of self-worth alongside dominant and cold interpersonal behavior (Buss & Chiodo, 1991; Miller, Lynam, Siedor, et al., 2018), which is in line with the concept of grandiose narcissism as studied in social/personality and clinical psychology. Grandiose narcissism as a trait was devised from the diagnostic criteria for Narcissistic Personality Disorder (NPD) in the third edition of the *Diagnostic and Statistical Manual of Mental Disorders* (3rd ed.; *DSM-III*; American Psychiatric Association [APA], 1980), which Raskin and Hall (1979, 1981) translated into self-report items in the Narcissistic Personality Inventory (NPI). It encompasses statements such as “I think I am a special person” or “I am more capable than other people” (Raskin & Terry, 1988). NPI grandiose narcissism can be characterized mainly by high extraversion and low agreeableness in the FFM (e.g., Ackerman et al., 2011), paralleling the notion of highly narcissistic individuals as “disagreeable extraverts” (Paulhus, 2001, p. 228). While the NPI has long been the standard measure of trait grandiose narcissism and is still being extensively used, one of its problems is that the total score intermingles agentic and antagonistic aspects of narcissism (Back et al., 2013), which have substantially different nomological networks (see below).

Newer models conceptualize grandiose narcissism along intrapersonal and interpersonal dynamics and ground them in broad personality models: Morf and Rhodewalt (2001) describe Narcissism in terms of the dynamic processes involved in the construction and maintenance of a grandiose self—the central self-regulatory goal in narcissism that is played out in the social arena (see also Morf et al., 2011). Building on this line of thought, in the narcissistic admiration and rivalry concept (NARC), Back and colleagues (2013) describe characteristic strategies of either self-enhancement or self-protection which serve to maintain this grandiose self. Viewed from an individual differences perspective, these strategies reflect in either more agentic or more antagonistic patterns of experience and behavior (Back, 2018; see also Grapsas et al., 2020). These agentic and antagonistic aspects, respectively, can be thought of as narcissistic variants of broad FFM dimensions, which served as the theoretical rationale for the construction of the FFNI (Glover et al., 2012), as discussed in more detail shortly.

#### Vulnerable Narcissism

Complemental to the study of grandiose narcissism, vulnerable narcissism was studied in terms of a largely independent trait in the past decades (Fossati et al., 2009). While the concept of narcissistic vulnerability had long been part of clinical theories (Pincus & Lukowitsky, 2010), Wink (1991) noted in nonclinical factor-analytic studies of several narcissism scales that they sort into two largely orthogonal dimensions which he called “grandiosity-exhibitionism” and “vulnerability-sensitivity” (p. 590). Whereby the first of these aligns with the construct of grandiose narcissism, Wink’s “second face” of narcissism constitutes the independent yet related construct of vulnerable narcissism^1^ in terms of “introversion, defensiveness, anxiety, and vulnerability to life’s traumas” (p. 590). Despite their opposing experiential and behavioral tendencies, Wink noted both share “common narcissistic characteristics of conceit, self-indulgence, and disregard for the needs of others” (p. 596), thereby already anticipating contemporary personality models (see below). Building on this distinction, Hendin and Cheek (1997) constructed the Hypersensitive Narcissism Scale (HSNS) for the assessment of vulnerable narcissism. It encompasses items such as “I can become entirely absorbed in thinking about my personal affairs, my health, my cares or my relations to others” (p. 592). Individuals scoring high on the HSNS are characterized by neuroticism, disagreeableness, and introversion in the FFM (Jauk et al., 2017; Miller et al., 2011). Although the HSNS is a widely used measure of vulnerable narcissism and is valid with respect to expert ratings (Miller et al., 2014), a shortcoming is that—as to the NPI—it intermingles discernible aspects of vulnerable narcissism which have substantially different nomological networks, in this case, antagonistic and neurotic aspects (Miller et al., 2011).

### The Structure of Narcissism

As outlined above, both grandiose and vulnerable narcissism draw upon core narcissistic features such as self-importance, but, beyond that can be characterized by other trait constellations. In their Narcissism Spectrum Model, Krizan and Herlache (2018) formalized this relationship by proposing a spectrum of narcissism ranging from grandiosity to vulnerability, with entitlement and self-importance standing at the core, and general approach- or avoidance motivation shaping this common core either in the direction of grandiosity or vulnerability. The model receives support from factor-analytic studies of different narcissism measures (Krizan & Herlache, 2018) and relations of grandiose and vulnerable narcissism with measures of behavioral activation and inhibition indicating either bold or reactive interpersonal styles (Spencer et al., 2018).

Similarly, in their Trifurcated Model of narcissism grounded in the FFM, Miller and colleagues (2016, 2017; Weiss et al., 2019) assert that narcissism can be described by combinations of antagonism—standing at the core of both grandiosity and vulnerability—and agentic extraversion on one hand, and neuroticism on the other.^2^ The model is consistent with the process-oriented NARC model assuming self-enhancing self-regulatory strategies (agentic narcissism) and self-protecting self-regulatory strategies (antagonistic narcissism; Back et al., 2013; see also Back, 2018) as central means to the maintenance of a grandiose self. The NARC has been expanded to encompass neurotic aspects of narcissism, which are conceptualized as an “exit strategy” when neither agentic nor antagonistic strategies are successful in maintaining or restoring a grandiose self (Back, 2018). Of note, though expressed in different vocabularies, the Narcissism Spectrum Model and the Trifurcated Model are highly similar and consistent in their predictions (Wright & Edershile, 2018), and together with the process-oriented NARC model build the state of the art of personality models on narcissism. Beyond that and importantly, the Trifurcated Model not only offers an alternative description of grandiose and vulnerable narcissism along the FFM dimensions, but also—together with the NARC—an extended conceptualization of narcissism as it discerns agentic, antagonistic, and neurotic aspects. The three-factor conceptualization can be thought of as a more nuanced decomposition of grandiose and vulnerable narcissism (Crowe et al., 2019). This is not only meaningful for personality research as it helps to understand which factors underlie associations with different criteria, but also for the clinical diagnosis of narcissism and other personality disorders, which currently exhibits a shift from traditional categorical approaches to the description of personality disorders in terms of dimensional (FFM-based) profiles (Bender et al., 2011; Tyrer et al., 2019). Case studies of NPD vividly demonstrate the viability of this approach as individuals who would otherwise receive the same diagnosis can vary considerably on pathological FFM traits (Pincus et al., 2016). Dimensional and hierarchical models of narcissism thus not only aid the scientific understanding of the multifaceted construct, but also offer new diagnostic perspectives.

### The FFNI

The FFNI (Glover et al., 2012) is the to date most comprehensive inventory for the assessment of narcissism from an FFM perspective, encompassing both its grandiose and vulnerable aspects (two-factor model) as well as its agentic, antagonistic, and neurotic aspects (three-factor model). The construction rationale of the FFNI is the assumption that complex personality configurations, such as narcissism, can be described by (narcissistic) variants of corresponding FFM facets and traits, paralleling works on psychopathy (Lynam et al., 2011) or Machiavellianism (Collison et al., 2018). For instance, the *entitlement* facet (antagonistic narcissism) was constructed as a narcissistic variant of the FFM *altruism* facet (agreeableness); a corresponding item is “it may seem unfair, but I deserve extra (i.e., attention, privileges, rewards)” (Glover et al., 2012). Similarly, the *authoritativeness* facet (agentic narcissism) was constructed as a narcissistic variant of the FFM *assertiveness* facet (extraversion); a sample item is “I am comfortable taking on positions of authority.” Altogether, the FFNI comprises 15 facets which were selected based on prior expert ratings and meta-analyses (see Table 2 and Glover et al., 2012). In addition to the original 148-item long form of the FFNI (FFNI-LF), a 60-item short form (FFNI-SF) was later constructed (based on Item Response Theory; Sherman et al., 2015). Most recently, a 15-item Super-Short Form of the FFNI (FFNI-SSF) was constructed using the highest-loading item of each facet (Packer West et al., 2021).

#### Convergent Validity

The FFNI has been found to be a reliable and valid measure of different aspects of narcissism. Its grandiose aspects correlate highly (*r* = .74) with other trait measures of grandiose narcissism such as the NPI (Raskin & Hall, 1979) but also with clinically oriented self-report measures of NPD (.55 ≤ *r* ≤ .60) such as the Minnesota Multiphasic Personality Inventory (MMPI; Morey et al., 1985) or Personality Diagnostic Questionnaire (PDQ; Bagby & Farvolden, 2004; see initial publication by Glover et al., 2012). Similarly, its vulnerable aspects correlate substantially with the HSNS (Hendin & Cheek, 1997) as a trait measure of vulnerable narcissism (*r* = .65) and the vulnerable subscale of the Pathological Narcissism Inventory (*r* = .74; PNI; Pincus et al., 2009; see Glover et al., 2012). Grandiose as well as vulnerable aspects are related to *DSM*-based ratings of NPD (grandiose: .45 ≤ *r* ≤ .64, vulnerable: .24 ≤ *r* ≤ .36; Miller, Few, Wilson, et al., 2013). For the FFNI-SF, similar validity coefficients were observed across several samples, for instance with the NPI (.62 ≤ *r* ≤ .73) and the HSNS (.59 ≤ *r* ≤.71; Sherman et al., 2015). The FFNI-SF factors further show correlations with the grandiose (.27 ≤ *r* ≤ .55) and vulnerable (.70 ≤ *r* ≤ .82) subscales of the PNI (Fossati et al., 2018; Sherman et al., 2015). As predicted by the Narcissism Spectrum Model (Krizan & Herlache, 2018), both grandiose and vulnerable narcissism show substantial correlations with psychological entitlement (grandiose: *r* = .54, vulnerable: *r* = .33; Hart et al., 2020). The FFNI also shows discriminant validity across studies and samples, as grandiose and vulnerable aspects are unrelated or only moderately related to measures such as the HSNS or the NPI in crossed-over analyses (grandiose: .16 ≤ *r* ≤ .36, vulnerable: −.07 ≤ *r* ≤ .12; Glover et al., 2012; Sherman et al., 2015).

The FFNI grandiose and vulnerable factors are both associated with other socially aversive personality traits, among them the “Dark Triad” traits Machiavellianism and psychopathy. Correlations with these traits are substantial in full-scale measures (grandiose: .42 ≤ *r* ≤ .64, vulnerable: .41 ≤ *r* ≤ .57; Miller, Gentile, & Campbell, 2013) and short measures (Short Dark Triad, SDT; Jones & Paulhus, 2014; .76 ≤ *r* ≤ .84; Wehner et al., 2021), and substantial even when variance of the other two traits is partialed out (.31 ≤ *r* ≤ .41; Somma et al., 2020). FFNI vulnerability displays lower correlations to SDT narcissism (.11 ≤ *r* ≤ .40; Wehner et al., 2021).

As outlined above, the FFNI scales can not only be described at the two-factor level of grandiose and vulnerable narcissism, but also at the three-factor level of agentic, antagonistic, and neurotic narcissism from an FFM perspective. Within this framework, the FFNI factors display the expected pattern of correlations with FFM measures (absolute *r*s from .51 to .61; Miller et al., 2016; Rogoza et al., 2021), and they align conceptually with the distinction between admiration and rivalry (Back, 2018). Evidence also points to the expected relations with admiration and rivalry (from the Narcissistic Admiration and Rivalry Questionnaire, NARQ; Back et al., 2013) on an empirical basis (admiration: .53 ≤ *r* ≤ .70, rivalry: .63 ≤ *r* ≤ .71; Miller et al., 2016; Rogoza et al., 2021). The FFNI further displays expected convergent validity with related FFM traits at facet level, with relations being stronger for clinical measures (selected facets .63 ≤ *r* ≤ .83 with the Personality Inventory for *DSM-5*, PID-5; *Diagnostic and Statistical Manual of Mental Disorders* (5th ed.; *DSM-5*; APA, 2013; Krueger et al., 2012)) than for nonclinical measures (selected facets’ absolute correlations ranging between .35 ≤ *r* ≤ .75 with the NEO-Personality Inventory—Revised, NEO-PI-R; Costa & McCrae, 1992; see Helle & Mullins-Sweatt, 2019).

#### Nomological Network

Regarding the nomological network of the FFNI in other aspects of personality, interpersonal experience and behavior, and psychological functioning, one of the most general notions is that grandiose narcissism—particularly its agentic aspects—is related to approach motivation (Spencer et al., 2018) and egotistical, but—from the perspective of the individual—largely adaptive functioning (cf. Kaufman et al., 2020), whereas vulnerable narcissism—particularly its neurotic aspects—is related to avoidance motivation (Spencer et al., 2018) and a wide array of problematic patterns of experience and behavior, psychopathological symptoms, and generally reduced personality functioning (reduced self- and interpersonal functioning; APA, 2013; for data across different inventories, see Fossati et al., 2018; Miller, Lynam, Vize, et al., 2018). This is evident, for instance, in positive associations of FFNI grandiose narcissism with self-reported mental toughness (Papageorgiou, Gianniou, et al., 2019), status pursuit (Zeigler-Hill et al., 2021), self-reported social success (Oltmanns et al., 2018), hubristic but also authentic pride (Kaufman & Jauk, 2020), negative associations with experiencing feelings of guilt (Kaufman et al., 2020), fear (Jauk & Kaufman, 2018), or depression (Papageorgiou, Denovan, & Dagnall, 2019), and positive associations with adaptive coping, self-esteem (due to its agentic aspects; Jauk & Kaufman, 2018; Miller et al., 2016), and life satisfaction (again due to agentic aspects; Kaufman et al., 2020). FFNI grandiose narcissism is unrelated to explicit negative views of the self or others (Kaufman et al., 2020), unrelated (Kaufman et al., 2020), or weakly to moderately related to attachment anxiety and avoidance (Miller, Gentile, & Campbell, 2013), but its antagonistic aspects do relate to negative views of the self and others, a cynical-distrustful social attitude, and attachment anxiety and avoidance (Kaufman et al., 2020; Szymczak et al., 2020). Consequently, the FFNI grandiose dimension is associated with a cold-dominant interpersonal style (peaking in the arrogant-calculating octant of the interpersonal circumplex; Miller, Gentile, & Campbell, 2013), which can be hypothesized to draw more upon its antagonistic aspects, and game-playing (ludus) and obsessive (mania) love styles (Miller, Gentile, & Campbell, 2013). Also, FFNI grandiose narcissism—particularly its antagonistic aspects—is related to self-reports of anger, aggression, and externalizing symptoms (Sauls & Zeigler-Hill, 2020; Vize et al., 2019, 2020), laboratory-based measures of aggression (Chester & Lasko, 2019; Hyatt et al., 2019) or measures of racial prejudice (Infante et al., 2019). This makes the FFNI—particularly FFNI antagonistic narcissism—a potentially relevant predictor in forensic psychology and criminology (cf. DeLisi, 2019), which is supported by recent evidence showing that antagonism in general (Niemeyer et al., 2022) and antagonistic narcissism specifically (Niemeyer et al., 2021) are highly relevant predictors of criminal behavior.

FFNI vulnerable narcissism, on the contrary, shows associations with experiencing less positive and more negative affect (Jauk & Kaufman, 2018), experiencing shame (Di Sarno et al., 2020), lower authentic and higher hubristic pride (Kaufman & Jauk, 2020), higher attachment anxiety and avoidance (particularly its neurotic aspects; Kaufman et al., 2020; Miller, Gentile, & Campbell, 2013), feelings of social exclusion (Mazinani et al., 2021), a cold-submissive interpersonal style (peaking in the aloof-introverted octant of the interpersonal circumplex; Miller, Gentile, & Campbell, 2013), which can be hypothesized to relate particularly to its neurotic aspects. FFNI vulnerable narcissism further relates to game-playing (ludus) as well as obsessive (mania) love styles (like the grandiose dimension; Miller, Gentile, & Campbell, 2013) and pathologically altruistic behavior (Kaufman & Jauk, 2020). It is associated with interpersonal problems and social dysfunction (Fossati et al., 2018; Oltmanns et al., 2018) and a wide array of psychopathology (particularly internalizing pathology such as anxiety or depression; Jauk & Kaufman, 2018; Kaufman et al., 2020; Miller, Gentile, & Campbell, 2013; Papageorgiou, Denovan, & Dagnall, 2019). Regarding psychological adjustment, FFNI vulnerable narcissism is associated with lower self-esteem (due to its neurotic aspects; Jauk & Kaufman, 2018; Miller et al., 2016) and lowered well-being (as evident in lower life satisfaction, feelings of purpose, or self-acceptance, for instance, which are mainly due to neurotic, but also antagonistic aspects; Kaufman et al., 2020). Like grandiose narcissism, FFNI vulnerable narcissism is related to aggression, though associations are mainly driven by its antagonistic aspects (Vize et al., 2019). Ultimately, FFNI vulnerable narcissism is related to suicidal ideation (Brioschi et al., 2020).

While FFNI grandiose narcissism is broadly associated with egotistical-adaptive functioning from the perspective of the individual, it is also associated with feelings of alienation, a weak sense of self, maladaptive defense, and dissociation (Kaufman et al., 2020; Şar & Türk-Kurtça, 2021), reports of childhood trauma (Şar & Türk-Kurtça, 2021), and substance use (Miller, Gentile, & Campbell, 2013; Welker et al., 2019). Also, when contrasting self- and informant-reports, it becomes evident that others do not see grandiose narcissism as favorably as highly grandiose individuals do themselves (despite generally high agreement on grandiosity between self and informants; Oltmanns et al., 2018).

### Present Research

We construct and validate a German version of the FFNI and a novel brief version. In Study 1, we construct a German translation of the FFNI and investigate its factor structure in a confirmatory framework. Next, we use a comprehensive battery of validation measures to investigate the nomological network of the FFNI regarding other narcissism scales, neighboring socially aversive personality traits, general FFM measures, as well as measures of interpersonal experience and behavior and adaptive and maladaptive psychological functioning. In Study 2, we construct a 30-item brief form of the FFNI which optimizes its internal structure and external validity using ACO. Results of supplementary analyses as well as data, and statistical code needed to reproduce all main and supplementary analyses are provided at https://osf.io/ae7vr/.

## Study 1: Development and Validation of the German FFNI

### Aims and Hypotheses

The main aims of this study are to (a) construct a German version of the FFNI, (b) test its factor structure at different hierarchical levels in a confirmatory framework, and (c) corroborate and extend its nomological network regarding different aspects of personality and psychological functioning, (d) complemented by analyses of clinically relevant criteria and groups.

The FFNI has been adapted for the use in Italian (Fossati et al., 2018), Norwegian (only vulnerable subscales; Prendergast et al., 2019), Polish (Rogoza et al., 2021), Russian (Papageorgiou et al., 2021), and Japanese (Dai et al., 2021) languages. However, there is no validated German version of the FFNI, despite a significant amount of research on narcissism stemming from German-speaking countries. We aim to fill this gap by providing a validated German FFNI. Second, though the factor structure of the FFNI has a solid foundation in the FFM and is compatible with process-based models of narcissism (Back, 2018), only a limited number of studies directly investigated its factor structure, yielding heterogeneous results (Dai et al., 2021; Fossati et al., 2018; Miller et al., 2016; Papageorgiou et al., 2021; Rogoza et al., 2021; Sherman et al., 2015). Among those, only a single study investigated the factor structure in a confirmatory framework (Papageorgiou et al., 2021). Moreover, factor analyses were performed either only for the FFNI-SF (Dai et al., 2021; Fossati et al., 2018; Papageorgiou et al., 2021; Rogoza et al., 2021) or, within the FFNI-LF, only at facet- but not item-level (Miller et al., 2016; Sherman et al., 2015). Hence, our second aim is to investigate the factor structure in a confirmatory framework at different hierarchical levels including both the item and facet level.

As our third aim, we replicate and extend findings on the nomological network of the FFNI using other narcissism scales, measures of neighboring socially aversive personality traits, general FFM measures, as well as measures of interpersonal experience and behavior and adaptive and maladaptive psychological functioning. The latter are complemented by analyses of FFNI profiles related to clinically relevant characteristics (prior diagnoses/treatment) and groups (forensic sample).

On the basis of the reviewed literature, we hypothesize that the FFNI two- and three-factor model scores will show substantial convergent validity with measures of grandiose and vulnerable narcissism (NPI, HSNS) and agentic as well as antagonistic narcissism (NARQ admiration vs. rivalry and NPI leadership authority, grandiose exhibitionism vs. entitlement/exploitativeness). We further hypothesize to find substantial convergence with the PNI, though in this case, we expect stronger alignment of the vulnerable than the grandiose dimensions, as PNI grandiosity, on a conceptual basis, puts stronger weight on maladaptive aspects (Pincus et al., 2009; see also Krizan & Herlache, 2018). Both, grandiose and vulnerable narcissism should be associated with psychological entitlement (though grandiose narcissism to a stronger extent), and correlations should be highest with antagonistic narcissism in the three-factor model. FFNI grandiose and to some extent vulnerable narcissism are expected to display correlations with socially aversive personality traits (see above).

The FFNI three-factor model dimensions should show substantial associations with the respective dimensions from general FFM inventories. Based on the literature, higher correlations can be expected for clinical than nonclinical FFM inventories. However, this concerns only the PID-5 dimensions of antagonism and neuroticism, as the PID-5 intro-/extraversion dimension emphasizes maladaptive introversion (detachment; Krueger et al., 2012). For agentic narcissism, we thus expect stronger associations with a nonclinical measure of extraversion. Besides its associations with specific FFM traits, it can be hypothesized that primarily vulnerable (Miller, Lynam, Vize, et al., 2018) but also grandiose narcissism (Fossati et al., 2018) is accompanied by impaired personality functioning in terms of reduced self-regulatory and interpersonal capacities (with severe impairments indicating personality disorders; APA, 2013).

We further expect the German FFNI dimensions to show correlation patterns with measures of interpersonal experience and behavior as well as psychological adjustment and maladjustment which are in accordance with the reviewed literature. In particular, grandiose narcissism should be associated with general approach motivation (particularly its agentic aspects), hubristic pride (and, to a lesser extent, also authentic pride), a cold-domineering interpersonal style (particularly its antagonistic aspects), game-playing and obsessive love styles, and measures of anger and aggression (particularly its antagonistic aspects). Antagonistic aspects should further relate to attachment anxiety and avoidance, while no particularly strong associations are expected for overall grandiose narcissism. Grandiose narcissism, particularly its agentic aspects, should relate positively to measures of psychological adjustment (self-esteem, life satisfaction) and negatively to measures of maladjustment/psychopathology. Vulnerable narcissism should be associated with avoidance orientation (particularly neuroticism), hubristic pride, a wide array of interpersonal problems, game-playing and obsessive love styles, anger, and aggression (mainly antagonistic aspects), and attachment anxiety and avoidance (particularly neurotic aspects). It should correlate negatively with adaptive adjustment (self-esteem and life satisfaction) and positively with maladjustment (e.g., symptom load). These correlations should be mostly due to neurotic, but also antagonistic aspects.

We complement validity analyses of dimensional measures of maladaptive adjustment by more objective clinical and forensic criteria. For this, we assessed prior diagnoses in two of the included samples and administered the German FFNI in a sample of incarcerated young criminal offenders. Based on the literature, we expect that individuals with prior diagnoses will show elevated scores on vulnerable narcissism, particularly its neurotic aspects, and criminal offenders will show elevated scores on antagonistic narcissism. None of our hypotheses were preregistered. We report how we determined our sample size, all data exclusions, all manipulations, and all measures in the study.

### Method

#### Samples

We gathered data from eight samples of German-speaking participants, four of which completed the long, and four of which the short version of the FFNI alongside different validity measures. The rationale was to pool several samples to obtain the largest possible sample size for analyses of the factorial structure and validity of the FFNI (see Analysis Plan). Altogether, the samples comprised *N* = 2,921 participants (2,140 women, 778 men, 3 other) with a mean age of 25.59 years (*SD* = 7.67). Among those, *N* = 1,823 completed the long, and *N* = 1,098 the short FFNI. All samples were convenience samples assessed online, with the exception of sample 8, which includes young criminal offenders incarcerated in a correctional institution in the German state of Mecklenburg-Vorpommern. All data were collected in accordance with the relevant local guidelines and regulations and with the Declaration of Helsinki. There were no missing data (incomplete data sets were excluded a-priori). Beyond the scales listed in Table 1, further personality scales which are not analyzed here (due to redundancy or nonrelevance to the research questions) were administered in some samples.

**Table 1.** Samples and Scales.

Sample	Country	FFNI vers.	*N*	m/w/o	*M* (*SD*) age	Validity measures
1	CH/DE	LF	433	98/335/0	25.64 (7.48)	NEO-PI-R, RSES
2	CH/DE	LF	463	94/369/0	25.78 (7.61)	NPI, NARQ, HSNS, PNI, DAPP, PES, SDT, MSI-BPD, RSES
3	CH/DE	LF	465	101/364/0	25.37 (7.15)	BIS/BAS, IIP-32, ECR-RD12, BPAQ, LAS, RSES
4	CH/DE	LF	462	97/365/0	26.21 (7.13)	AHPS, PID-5, RSES, SWLS, CES-D
5	DE	SF	564	156/408/0	24.94 (5.98)	NPI, NARQ, HSNS, PES, ECR-RD12, RSES
6	DE	SF	258	94/164/0	24.62 (5.33)	Self-Reported Diagnoses, BSI
7	DE	SF	208	70/135/3	31.03 (13.38)	Self-Reported Diagnoses, IPO, BSI
8	DE	SF	68	68/0/0	20.46 (3.14)	Forensic Sample, IPO

*Note*. FFNI = Five-Factor Narcissism Inventory; CH = Switzerland; DE = Germany; LF = long form; SF = short form; NEO-PI-R = NEO-Personality Inventory-Revised; RSES = Rosenberg Self-Esteem Scale; NPI = Narcissistic Personality Inventory; NARQ = Narcissistic Admiration and Rivalry Questionnaire; HSNS = Hypersensitive Narcissism Scale; PNI = Pathological Narcissism Inventory; DAPP = Dimensional Assessment of Psychopathology; PES = Psychological Entitlement Scale; SDT = Short Dark Triad; MSI-BPD = McLean Screening Instrument for Borderline Personality Disorder; BIS/BAS = Behavioral Inhibition/Behavioral Activation Scales; IIP-32 = Inventory of Interpersonal Problems-32; ECR-RD12 = Experience in Close Relationships-Revised Questionnaire; BPAQ = Buss-Perry Aggression Questionnaire; LAS = Love Attitudes Scale; AHPS = Authentic Hubristic Pride Scale; PID-5 = Personality Inventory for *DSM*-5; SWLS = Satisfaction with Life Scale; CES-D = Center for Epidemiologic Studies Depression Scale; BSI = Brief Symptom Inventory; IPO = Inventory of Personality Organization.

Among the pooled sample, *N* = 1,745 (59.74%) of participants were students, 475 (16.26%) were employed, 50 (1.71%) were apprentices or secondary / high school pupils, 90 (3.08%) retired or unemployed, and the 68 (2.33%) participants of sample 8 were incarcerated (information on vocational status was unavailable for 493 participants, or 16.88% of the pooled sample). Table 1 provides detailed characteristics of the individual samples and the measures included therein. Self-reports on previously professionally diagnosed mental disorders were available in samples 6 and 7. Of the 466 participants in those samples, 363 (77.90%) reported no history of mental disorder, 55 (11.80%) reported a past disorder, and 48 (10.30%) a present disorder.

#### FFNI

The FFNI is a multidimensional measure of narcissism grounded in the FFM. It comprises 148 items (FFNI-LF)/60 items (FFNI-SF) belonging to 15 facets which were constructed as narcissistic variants of FFM facets (for examples, see introduction; Glover et al., 2012). Within the two-factor model, these facets can be summarized to reflect factors of grandiose narcissism (*indifference*, *exhibitionism*, *authoritativeness*, *grandiose fantasies*, *manipulativeness*, *exploitativeness*, *entitlement*, *lack of empathy*, *arrogance*, *acclaim seeking*, *thrill seeking*) and vulnerable narcissism (*reactive anger*, *shame*, *need for admiration*, *distrust*; Glover et al., 2012). Within the three-factor model, the FFNI provides factor scores of agentic narcissism (*acclaim seeking*, *authoritativeness*, *grandiose fantasies*, *exhibitionism*), antagonistic narcissism (*manipulativeness*, *exploitativeness*, *entitlement*, *lack of empathy*, *arrogance*, *reactive anger*, *distrust*, *thrill seeking*), and neurotic narcissism (*shame*, *indifference* [reversed], *need for admiration;*Miller et al., 2016) (see Note 2).

The FFNI was initially translated into German independently by several members of the research groups from Bern and Münster. Next, a final consensus version was created by three psychologists all experts in narcissism and one of whom is a bi-lingual native speaker. This process involved an iterative approach in which each translated item was evaluated and in case of deviant translations the best wording was determined through discussion until consensus was reached. Following this, a back-translation was performed by a different bi-lingual native speaker, upon which any discrepancies again were resolved through discussion. Items of the German FFNI are available in Supplemental Table S1 and on the Open Science Framework (OSF): https://osf.io/ae7vr/

#### Validity Measures

This section provides information on the validity measures included in this study. Table 1 shows the samples in which the measures were used, Table 4 displays internal consistencies.

##### Narcissism and Related Constructs

###### NPI

The NPI (Raskin & Hall, 1979; German version by Schütz et al., 2004) is a 40-item forced choice measure of grandiose narcissism, covering particularly its agentic but also its antagonistic aspects (Krizan & Herlache, 2018). Beyond the general score, we used the factors extracted by Ackerman and colleagues (2011) as indicators of agentic (*leadership/authority* and *grandiose exhibitionism*) and antagonistic narcissism (*entitlement/exploitativeness*^3^; Ackerman et al., 2011, p. 69).

###### NARQ

The NARQ is an 18-item measure of agentic (admiration) and antagonistic (rivalry) aspects of grandiose narcissism (Back et al., 2013). The admiration dimension encompasses the facets *grandiosity*, *uniqueness*, and *charmingness*, the rivalry dimension encompasses the facets *devaluation*, *supremacy* and *aggressiveness*.

###### Hypersensitive Narcissism Scale (HSNS)

The HSNS is a 10-item measure of vulnerable narcissism covering its antagonistic and neurotic aspects (for details, see introduction; Hendin & Cheek, 1997; German translation by the authors, see also Morf et al., 2017).

###### Pathological Narcissism Inventory (PNI)

The PNI is a 54-item measure of pathological narcissism (Pincus et al., 2009; German version by Morf et al., 2017). It encompasses grandiose and vulnerable subscales, though the grandiose scales put an emphasis on maladaptive aspects (Pincus et al., 2009) and are in this regard different from trait measures of grandiose narcissism (Krizan & Herlache, 2018). The grandiose subscales of the PNI cover *exploitativeness*, *grandiose fantasy*, *self-sacrificing self-enhancement*, and *entitlement rage*.^4^ The vulnerable subscales cover *contingent self-esteem*, *hiding the self*, *devaluing* (Pincus et al., 2009).

###### Dimensional Assessment of Psychopathology—Basic Questionnaire (DAPP-BQ)

We used the DAPP-BQ (Livesley & Jackson, 2009; German version by Angleitner et al., 2011) 16-item narcissism scale as clinically oriented measure of narcissism.

###### Psychological Entitlement Scale (PES)

We used the nine-item PES (Campbell et al., 2004; German translation by Morf et al., 2017) as a measure of entitlement, which is regarded an individual difference construct standing at the core of narcissism (see introduction).

###### Short Dark Triad (SDT)

The Short Dark Triad (Jones & Paulhus, 2014; German version by Wehner et al., 2021) assesses narcissism, Machiavellianism, and psychopathy with nine items each.

##### General Personality Measures

###### NEO Personality Inventory—Revised (NEO-PI-R)

The NEO-PI-*R* (Costa & McCrae, 1992; German version by Ostendorf & Angleitner, 2004) is a comprehensive 240-item inventory assessing the FFM dimensions and their facets.

###### Behavioral Inhibition/Behavioral Activation Scales (BIS/BAS)

The BIS/BAS (Carver & White, 1994; German version by Strobel et al., 2001) is a 24-item personality measure grounded in Gray’s reinforcement sensitivity theory (e.g., Gray, 1970) and assesses the individuals’ general approach and avoidance motivation.

###### Authentic Hubristic Pride Scale (AHPS)

The AHPS (Tracy & Robins, 2007; German version by Sullivan, 2010) measures the respective constructs with seven items each; particularly hubristic pride (i.e., positive attributions to the global self rather than one’s actions; Tracy & Robins, 2007) has been associated with narcissism (Tracy et al., 2009).

##### Clinical Personality Measures

###### Personality Inventory for *DSM*-5 (PID-5)

The PID-5 (Krueger et al., 2012; German version by Zimmermann et al., 2014) was used to assess maladaptive variants of the FFM traits as conceptualized in the *DSM-5* (APA, 2013).

###### McLean Screening Instrument for Borderline Personality Disorder (MSI-BPD)

The MSI-BPD (Zanarini et al., 2003; German version Kröger et al., 2010 [translation by S. Hörz]) screens for the respective personality disorder as defined in the *Diagnostic and Statistical Manual of Mental Disorders* (4th ed.; *DSM-IV*; APA, 1994) using 10 dichotomous items.

###### Inventory of Personality Organization (IPO)

The IPO (Lenzenweger et al., 2001; German 16-item version by Zimmermann et al., 2013) assesses the level of personality organization on the three dimensions *identity diffusion* (weakly integrated concept of self and significant others), *primitive defense* (reactions to self-threats such as projection or splitting), and impaired *reality testing* (capacity to differentiate the inner from the outer stimuli; Lenzenweger et al., 2001). The IPO is conceptually similar to the A-criterion of the *DSM-5* Alternative Model for Personality Disorders and can be conceived a measure of general personality functioning (Zimmermann et al., 2015).

##### Interpersonal Experience and Behavior

###### Inventory of Interpersonal Problems (IIP-32)

The IIP (Horowitz et al., 1988, German 32-item version IIP-32 by Thomas et al., 2011) assesses interpersonal problems in octants of the interpersonal circumplex model. Internal consistencies of the single scales are in the expected range given that each scale has four items only (Thomas et al., 2011). The IIP scales can be collapsed into a single score indicating the overall amount of interpersonal problems.

###### Experience in Close Relationships—Revised Questionnaire (ECR-RD12)

The 12-item German ECR-RD12 (Brenk-Franz et al., 2018; English original by Fraley et al., 2000) assesses attachment anxiety and avoidance with five and seven items, respectively.

###### Buss-Perry Aggression Questionnaire—Short Form (BPAQ-SF)

The 12-item BPAQ-SF (Diamond & Magaletta, 2006; German items by von Collani & Werner, 2005) assesses *anger* and *hostility* as well as *physical* and *verbal aggression*. A total score can be derived.

###### Love Attitudes Scale (LAS)

The LAS (Hendrick & Hendrick, 1986; German 60-item adaptation by Bierhoff et al., 1993) assesses the love styles *eros* (passionate love), *ludus* (game-playing love), *storge* (friendship love), *pragma* (pragmatic love), *mania* (obsessive love), and *agape* (altruistic love).

##### Psychological Adjustment and Maladjustment

###### Rosenberg Self-Esteem Scale (RSES)

We used the 10-item RSES (Rosenberg, 1965; German version by von Collani & Herzberg, 2003) for the assessment of self-esteem as an indicator of overall intrapersonal adjustment.

###### Satisfaction with Life Scale (SWLS)

We used the five-item SWLS (Diener et al., 1985; German version by Glaesmer et al., 2011) as an indicator of life satisfaction.

###### Brief Symptom Inventory (BSI)

We used the 53-item BSI (Derogatis & Melisaratos, 1983; German version by Franke, 2000, a short form of the Symptom Checklist (Derogatis & Lazarus, 1994) to assess overall symptom load (Global Severity Index, GSI) across the nine BSI scales.

###### Center for Epidemiologic Studies Depression Scale (CES-D)

We used the German version of the CES-D (Radloff, 1977), the 20-item “Allgemeine Depressionsskala” (Hautzinger & Bailer, 1993), to assess depressive symptoms.

#### Analysis Plan

Our rationale was to conduct analyses based on the largest set of available data across the samples. We pooled samples for analyses of reliability and factor structure for the different FFNI versions, leading to pooled data of *N* = 1,823 participants who filled in the FFNI-LF and *N* = 1,098 who filled in the FFNI-SF. Sample sizes for validity analyses differ depending on which inventories were included in the samples (see Tables 1 and 4). For analyses of internal consistency and factor structure of the short versions (novel brief form described in Study 2 and recently published FFNI-SSF), we used all available data of participants who filled in either the FFNI-LF or the FFNI-SF (*N* = 2,921).

The analysis proceeded in three steps—descriptives and internal consistencies, confirmatory examination of higher- and lower-order factor-structure, followed by convergent and discriminant validity. We first analyzed internal consistencies and intercorrelations of the German FFNI in its different versions (including the brief form presented in Study 2) at the two-factor, three-factor, and facet level. Next, we set up confirmatory factor analysis (CFA) models to analyze the factor structure at the different levels (correlated facets models, two-factor, and three-factor models). As the FFNI items display substantial cross-loadings, we complemented traditional CFA models with exploratory structural equation models (ESEMs; Asparouhov & Muthén, 2009). ESEMs combine the confirmatory with an exploratory factor analysis framework and allow for cross-loadings and rotation of solutions. We used target rotation to maximize the main loadings and minimize cross-loadings. As the ESEM technique does not directly allow the implementation of hierarchical models, we used the ESEM-within-CFA approach (Morin & Asparouhov, 2018) for models with more than two hierarchical levels (for a similar analysis strategy, see Morf et al., 2017).^5^ Here, the first-order factor loadings are first estimated in ESEM. These loadings (including cross-loadings) are then used as constraints for an estimation of the higher-order model in CFA. As not all item-level data were normally distributed, we attempted to estimate models using weighted least square mean and variance adjusted (WLSMV) estimators. However, many of the models could not be estimated using WLSMV (nonconvergence); therefore, we used maximum likelihood (ML) estimation for CFA and ESEM models instead.

Finally, we analyzed correlations with validity measures assessing narcissism and related constructs, nonclinical and clinical personality constructs, interpersonal experience and behavior, as well as psychological adjustment and maladjustment.

### Results and Discussion

#### Descriptive Statistics and Internal Consistencies

Table 2 shows the scale averages, internal consistencies, and intercorrelations of the FFNI (both two- and three-factor models constructed based on Glover et al., 2012 and Miller et al., 2016; see Method section) for the different versions. The scores of both models displayed high internal consistencies in the LF and SF (.95 ≤ α ≤ .89), the FFNI-SSF displayed somewhat lower but still good internal consistencies given its length (.60 ≤ α ≤ .73). At facet level, internal consistencies for the LF were between .70 ≤ α ≤ .89, those for the SF were between .69 ≤ α ≤ .90 (SSF has only one item per facet).

**Table 2.** Descriptive Statistics, Internal Consistencies, and Intercorrelations.

	*M* (*SD*), α				1				2				3				4				5			
Factor/Facet	LF (*N* = 1,823)	SF (*N* = 1,098)	BF (*N* = 2,921)	SSF (*N* = 2,921)	LF	SF	BF	SSF	LF	SF	BF	SSF	LF	SF	BF	SSF	LF	SF	BF	SSF	LF	SF	BF	SSF
Two-factor model																								
Grandiose Narcissism (1)	2.37 (0.41), .95	2.32 (0.52), .93	2.23 (0.53), .84	2.26 (0.54), .73																				
Vulnerable Narcissism (2)	2.82 (0.53), .91	2.89 (0.61), .83	2.97 (0.70), .72	2.60 (0.83), .60	.03	**.10**	**.13**	−.03																
Three-factor model																								
Agentic Narcissism (3)	2.85 (0.57), .93	2.82 (0.66), .87	2.70 (0.75), .76	2.78 (0.84), .68	**.83**	**.82**	**.82**	**.80**	.01	**.14**	**.20**	−.01												
Antagonistic Narcissism (4)	2.21 (0.41), .91	2.15 (0.52), .90	2.15 (0.53), .79	1.93 (0.54), .64	**.82**	**.88**	**.85**	**.86**	**.44**	**.38**	**.41**	**.16**	**.48**	**.56**	**.51**	**.43**								
Neurotic Narcissism (5)	3.19 (0.66), .94	3.30 (0.76), .89	3.32 (0.85), .84	3.36 (0.91), .70	**−.26**	**−.27**	**−.21**	**−.21**	**.83**	**.78**	**.79**	**.89**	**−.11**	−.03	.03	**−.07**	*.05*	**−.10**	−.04	**−.05**				
Facets																								
Reactive anger	2.62 (0.64), .77	2.59 (0.85), .76	2.90 (1.00), .70	—	**.30**	**.38**	**.34**	—	**.66**	**.60**	**.57**	—	**.26**	**.34**	**.32**	—	**.56**	**.56**	**.53**	—	**.33**	**.20**	**.16**	—
Shame	3.23 (0.80), .89	3.22 (0.90), .80	3.00 (1.05), .73	—	**−.23**	**−.14**	**−.11**	—	**.81**	**.80**	**.76**	—	**−.13**	.00	.01	—	**.07**	.03	**.06**	—	**.91**	**.87**	**.83**	—
Indifference	2.36 (0.73), .89	2.32 (0.91), .89	2.25 (0.92), .84	—	**.38**	**.42**	**.35**	—	**−.60**	**−.40**	**−.43**	—	**.13**	**.07**	*.04*	—	**.14**	**.30**	**.21**	—	**−.90**	**−.81**	**−.80**	—
Need for admiration	2.69 (0.69), .80	2.99 (0.88), .74	3.20 (1.07), .67	—	**−.07**	**−.14**	**−.08**	—	**.81**	**.78**	**.76**	—	−.03	−.02	**.11**	—	**.21**	.02	*.04*	—	**.86**	**.86**	**.87**	—
Exhibitionism	2.85 (0.73), .83	3.04 (0.90), .80	3.00 (0.95), .88	—	**.59**	**.54**	**.49**	—	**−.06**	.06	**.18**	—	**.75**	**.72**	**.67**	—	**.27**	**.32**	**.27**	—	**−.09**	.00	**.13**	—
Authorativeness	3.03 (0.73), .89	2.88 (0.90), .86	2.86 (0.99), .74	—	**.66**	**.63**	**.59**	—	**−.18**	*−.08*	−.02	—	**.74**	**.72**	**.67**	—	**.36**	**.41**	**.35**	—	**−.31**	**−.24**	**−.18**	—
Grandiose fantasies	2.55 (0.71), .84	2.28 (0.90), .78	2.24 (1.08), .75	—	**.71**	**.65**	**.65**	—	**.12**	.21	**.24**	—	**.82**	**.74**	**.78**	—	**.47**	**.50**	**.44**	—	.00	.03	**.10**	—
Manipulativeness	2.45 (0.66), .83	2.47 (0.91), .85	2.56 (0.97), .83	—	**.75**	**.70**	**.64**	—	**.10**	**.12**	**.11**	—	**.53**	**.54**	**.46**	—	**.71**	**.67**	**.62**	—	**−.12**	**−.11**	**−.10**	—
Exploitiveness	1.74 (0.66), .88	1.66 (0.79), .90	1.73 (0.88), .82	—	**.71**	**.69**	**.66**	—	**.25**	**.19**	**.18**	—	**.41**	**.41**	**.39**	—	**.81**	**.75**	**.71**	—	−.01	**−.08**	**−.05**	—
Entitlement	1.92 (0.55), .76	1.76 (0.74), .78	1.59 (0.80), .64	—	**.64**	**.65**	**.59**	—	**.24**	**.22**	**.21**	—	**.39**	**.41**	**.38**	—	**.71**	**.70**	**.64**	—	.03	−.05	.00	—
Lack of empathy	1.71 (0.58), .85	1.82 (0.75), .77	1.60 (0.74), .75	—	**.44**	**.57**	**.50**	—	**.17**	.02	.02	—	**.08**	**.22**	**.18**	—	**.61**	**.64**	**.57**	—	**−.06**	**−.30**	**−.21**	—
Arrogance	2.19 (0.55), .70	2.00 (0.75), .69	1.83 (0.77), .63	—	**.75**	**.74**	**.58**	—	**.14**	**.16**	**.13**	—	**.54**	**.49**	**.31**	—	**.73**	**.77**	**.63**	—	**−.09**	**−.18**	**−.13**	—
Acclaim seeking	2.96 (0.77), .88	3.07 (0.90), .81	2.69 (1.09), .67	—	**.60**	**.59**	**.65**	—	**.15**	**.22**	**.18**	—	**.77**	**.77**	**.79**	—	**.37**	**.42**	**.42**	—	*.06*	**.10**	*.05*	—
Thrill seeking	2.31 (0.81), .88	2.19 (0.94), .85	2.23 (1.10), .82	—	**.52**	**.60**	**.47**	—	.02	.02	.00	—	**.31**	**.38**	**.23**	—	**.51**	**.58**	**.50**	—	**−.14**	**−.19**	**−.15**	—
Distrust	2.75 (0.70), .79	2.75 (0.81), .70	2.78 (0.94), .60	—	**.14**	**.21**	**.24**	—	**.69**	**.61**	**.64**	—	−.03	**.08**	**.13**	—	**.53**	**.48**	**.54**	—	**.31**	**.21**	**.26**	—

*Note*. Correlations significant at p < .05 level are printed in italic, correlations significant at p < .01 are printed in bold. Two-factor model: the FFNI grandiose narcissism factor encompasses the facets *indifference*, *exhibitionism*, *authoritativeness*, *grandiose fantasies*, *manipulativeness*, *exploitativeness*, *entitlement*, *lack of empathy*, *arrogance*, *acclaim seeking*, and *thrill seeking*, the FFNI vulnerable narcissism factor encompasses *reactive anger*, *shame*, *need for admiration*, and *distrust* (Glover et al., 2012). Three-factor model: agentic narcissism comprises the facets *acclaim seeking*, *authoritativeness*, *grandiose fantasies*, and *exhibitionism*, antagonistic narcissism encompasses *manipulativeness*, *exploitativeness*, *entitlement*, *lack of empathy*, *arrogance*, *reactive anger*, *distrust*, and *thrill seeking*, neurotic narcissism encompasses *shame*, *indifference* (reversed), and *need for admiration* (Miller et al., 2016). LF = long form (Glover et al., 2012); SF = short form (Sherman et al., 2015);; BF = brief form (presented in Study 2); SSF = super-short form (Packer West et al., 2021); FFNI = Five-Factor Narcissism Inventory.

The intercorrelations among the scores according to the two- and three-factor models were generally as expected and similar for the different scale versions: grandiose and vulnerable narcissism displayed no or slight positive associations (for recent meta-analysis, see Jauk et al., 2021), grandiose narcissism was equally highly correlated with its constituent factors agentic narcissism and antagonistic narcissism and moderately negatively associated with neurotic narcissism. Vulnerable narcissism was unrelated or moderately related to agentic narcissism, moderately related to its constituent factor antagonistic narcissism (though lower in the SSF than the other versions), and strongly related to its constituent factor neurotic narcissism. Facet intercorrelations were, with few exceptions, highly similar for the different versions and can be seen in Table 2.

#### Factor Structure

To evaluate the factor structure of the German FFNI in a confirmatory framework, we tested CFA, ESEM, as well as ESEM-within-CFA models within the two- and three-factor structure. In the following, we discuss results for the FFNI-LF, -SF, and the -SSF here; results for the novel FFNI-BF are discussed in Study 2. Table 3 displays the complete fit statistics for the different models. Within the CFA models, fit was generally better for models taking the full hierarchical structure into account (factor -> facet -> item; models 4 & 7), compared to those which solely account for either the facet structure (factor -> facet; models 2 & 5) or item uniqueness (factor -> item; models 3 & 6). Within these hierarchical models, fit indices only partially indicated acceptable fit for the FFNI-LF in the two-factor (model 4: RMSEA =.047, CFI =.676, SRMR =.108) and three-factor solution (model 7: RMSEA = .046, CFI =.690, SRMR =.097). For the FFNI-SF, we observed less then optimal yet largely acceptable fit in the two-factor (model 4: RMSEA =.056, CFI =.821, RMSEA =.110) and in the three-factor solution (model 7: RMSEA =.052, CFI =.843, RMSEA =.092). For the SSF, hierarchical models cannot be estimated because facets are represented by single indicators. CFA models with items loading on factors did not generally display acceptable fit for this scale version in the two-factor (model 3: RMSEA =.101, CFI =.675, SRMR =.088) or three-factor solution (model 6: RMSEA =.077, CFI =.816, SRMR =.069). Fit was acceptable for this scale version, however, when using ESEM models as discussed next.

**Table 3.** Confirmatory Tests of Factor Structure.

		Long form (*N* = 1,823)				Short form (*N* = 1,098)				Brief form (*N* = 2,921)						Super-short form (*N* = 2,921)			
		Chi^2^ (*df*)	RMSEA, *p*_RMSEA<.05_	CFI	SRMR	Chi^2^ (*df*)	RMSEA, *p*_RMSEA<.05_	CFI	SRMR	Chi^2^ (*df*)	RMSEA, *p*_RMSEA<.05_	CFI	SRMR	Indicator loadings	Facet loadings	Chi^2^ (*df*)	RMSEA, *p*_RMSEA<.05_	CFI	SRMR
No.	CFA models																		
	Correlated facets																		
1	Facet→Item	48,021.472 (10,625)	.044, 1.000	.715	.079	5,427.948 (1,605)	.047, 1.000	.882	.063	1,218.923 (300)	0,032, 1.000	.970	.027	.42–.90		—	—	—	—
	Two-factor																		
2	Factor→Facet	5,652.607 (89)	.185, .000	.560	.148	2,078.637 (89)	.143, .000	.657	.133	4,584.172 (89)	.131, .000	.614	.121		.21–.78	—	—	—	—
3	Factor→Item	95,444.511 (10,729)	.066, .000	.356	.117	19,970.630 (1,709)	.099, .000	.438	.124	15,446.782 (404)	.113, .000	.501	.119	.19–.77		2,749.081 (89)	.101, .000	.675	.088
4	Factor→Facet→Item	53,332.713 (10,714)	.047, 1.000	.676	.108	7,494.203 (1,694)	.056, .000	.821	.110	6,012.535 (389)	.070, .000	.814	.102	.42–.91	.24–.97	—	—	—	—
	Three-factor																		
5	Factor→Facet	3,498.586 (87)	.147, .000	.730	.115	1,410.079 (87)	.118, .000	.772	.097	2,519.287 (87)	.098, .000	.791	.084		.32–.84	—	—	—	—
6	Factor→Item	No convergence				16,364.073 (1,707)	.088, .000	.549	.100	10,904.939 (402)	.095, .000	.652	.089	.25–.79		1,594.483 (87)	.077, .000	.816	.069
7	Factor→Facet→Item	51,408.833 (10,712)	.046, 1.000	.690	.097	6,788.347 (1,692)	.052, .001	.843	.092	3,651.130 (387)	.054, .000	.892	.072	.43–.92	.34–1.00	—	—	—	—
	ESEM models																		
	Correlated facets																		
8	Facet→Item	20,828.047 (8,763)	.027, 1.000	.908	.019	1,802.720 (975)	.028, 1.000	.975	.014	183.836 (105)	.016, 1.000	.997	.007	—^a^		—	—	—	—
	Two-factor																		
9	Factor→Facet	3,242.160 (76)	.151, .000	.750	.078	1,088.237 (76)	.110, .000	.826	.063	2,105.181 (76)	.096, .000	.826	.056		.26–.90	—	—	—	—
10	Factor→Item	80,773.290 (10,583)	.060, .000	.466	.077	16,556.838 (1,651)	.091, .000	.541	.083	11,337.561 (376)	.100, .000	.637	.072	.22–.97		1,466.861 (79)	.079, .000	.830	.050
11	ESEM within CFA (based on corr. fac. ESEM)	2,126.858 (8,852)	.028, 1.000	.906	.028	2,652.239 (1,064)	.037, 1.000	.951	.051	1,532.598 (180)	.051, .300	.955	.037	—^a^	—^a^				
	Three-factor																		
12	Factor→Facet	1,644.944 (63)	.117, .000	.875	.041	430.854 (63)	.073, .000	.937	.030	794.345 (63)	.063, .000	.937	.028		.25–.81	—	—	—	—
13	Factor→Item	65,453.667 (10,437)	.054, .000	.581	.056	12,888.655 (1,593)	.080, .000	.652	.060	7,945.993 (348)	.086, .000	.748	.052	—		487.140 (63)	.048, .788	.948	.023
14	ESEM within CFA (based on corr. fac. ESEM)	21,079.014 (8,850)	.028, 1.000	.907	.022	2,346.526 (1,062)	.033, 1.000	.960	.035	1,002.432 (178)	.040, 1.000	.973	.026	—^a^	—^a^				

*Note*. Models were estimated using maximum likelihood (ML) estimation. For details on modeling, see Study 1 methods. RMSEA = root mean square error of approximation; CFI = comparative fit index; SRMR = standardized root mean square residual; CFA = confirmatory factor analysis; ESEM = exploratory structural equation modeling.

a Parameter estimates are not displayed for models 8, 11, and 14 within the FFNI-BF because these involved out-of-bounds estimates which likely result from over-fitting an already optimized model (see Study 2, and OSF materials for full model information). Traditional hierarchical CFA models (models 4 & 7) seem sufficient for assessing the factor structure of the FFNI-BF and should be interpreted instead.

We proceeded the analysis with ESEM models, which allow for cross-loadings. These models generally outperformed traditional CFA models regarding model fit. Within the ESEM models, again, those taking the full hierarchical structure into account (ESEM-within-CFA; models 11 & 14) displayed the best data fit. Here, we observed good fit for the FFNI-LF in both the two-factor (model 11: RMSEA =.028, CFI =.906, SRMR =.028) and three-factor solution (model 14: RMSEA =.028, CFI =.907, SRMR = .022). Also, the FFNI-SF displayed good fit in the two-factor (model 11: RMSEA =.037, CFI =.951, SRMR =.051) and three-factor solution (model 14: RMSEA =.033, CFI =.960, SRMR =.035). Regarding the FFNI-SSF, ESEM models with cross-loadings of items on factors for the FFNI-SSF displayed largely acceptable fit in the two-factor solution (model 10: RMSEA =.079, CFI =.830, SRMR =.050) and good fit in the three-factor solution (model 13: RMSEA = .048, CFI =.948, SRMR =.023).

Taken together, though many of the models did not show acceptable fit by conventional standards, hierarchical CFA and ESEM models did so for different versions of the FFNI. Fit of ESEM-within-CFA models was generally superior to those of traditional higher-order CFA models, indicating that considering cross-loadings at item-level improves model fit. However, this also shows that the item sets of existing versions might not be optimally suited to capture the intended structure in an unidimensional manner. While ESEM models can effectively capture variance related to different aspects at item-level, we also note that they may run at the risk of over-fitting the data (all theoretically possible cross-loadings are being freely estimated). In Study 2, we thus developed a novel short form by selecting a set of items which best represented the intended structure of the FFNI in terms of model fit and factor loadings.

Turning to factor loadings (range displayed in Table 3, coefficients available in the OSF), within the evaluated models, all theoretically assumed (i.e., original subfactor assignment) loadings were significant, with the exceptions of the ESEM-within-CFA model (model 11) for the two-factor solution in the FFNI-LF, in which the facets *indifference* and *arrogance* did not load significantly on the grandiose narcissism factor, and the ESEM-within-CFA model for the three-factor solution (Model 14) in the FFNI-LF, in which the facets *arrogance*, *reactive anger*, and *distrust* did not load significantly on antagonistic narcissism. This indicates that considering cross-loadings at item-level, though improving model fit, unveils problems in the factorial structure of the FFNI-LF, in the way that these facets do no longer represent the designated higher-level factors (i.e., item-level variance can now be allocated to different facets). Such problems were not evident in the corresponding models of the FFNI-SF, where all facet loadings were significant, showing that shortened versions of the FFNI are better suited to capture the intended factor structure.

#### Criterion Validity

To investigate the criterion validity of the German FFNI with other measures of narcissism and related constructs, general as well as clinical personality measures, interpersonal experiences and behavior, as well as measures of psychological adjustment and maladjustment, we inspected correlations with the respective scales (see Table 4).

**Table 4.** Correlations With Validity Measures.

	α	FFNI vers.		Grandiose			Vulnerable			Agentic Narcissism			Antagonistic Narcissism			Neurotic Narcissism		
		LF	SF	LF/SF	BF	SSF	LF/SF	BF	SSF	LF/SF	BF	SSF	LF/SF	BF	SSF	LF/SF	BF	SSF
Narcissism and related constructs																		
NPI (*n* = 1,027)	.84	**·**	**·**	**.74**	**.69**	**.62**	−.05	−.04	**−.19**	**.73**	**.69**	**.65**	**.54**	**.47**	**.40**	**−.23**	**−.21**	**−.25**
Leadership/authority	.75			**.65**	**.60**	**.55**	*−.07*	*−.08*	**−.19**	**.65**	**.60**	**.60**	**.45**	**.39**	**.33**	**−.24**	**−.24**	**−.25**
Grandiose exhibitionism	.72			**.49**	**.45**	**.38**	**−.09**	*−.08*	**−.16**	**.52**	**.49**	**.45**	**.31**	**.26**	**.22**	**−.17**	**−.16**	**−.19**
Entitlement/exploitativeness	.41			**.45**	**.48**	**.45**	**.31**	**.28**	**.12**	**.36**	**.41**	**.35**	**.52**	**.50**	**.45**	**.14**	**.12**	*.07*
NARQ (*n* = 1,027)																		
Admiration	.86			**.72**	**.66**	**.63**	.01	.05	**−.08**	**.69**	**.65**	**.64**	**.54**	**.49**	**.43**	**−.15**	**−.12**	**−.15**
Grandiosity	.70			**.65**	**.61**	**.57**	−.02	.01	**−.10**	**.62**	**.59**	**.56**	**.48**	**.43**	**.40**	**−.18**	**−.15**	**−.17**
Strive for uniqueness	.68			**.56**	**.52**	**.50**	**.10**	**.14**	.01	**.56**	**.54**	**.53**	**.44**	**.40**	**.35**	−.03	.00	−.04
Charmingness	.76			**.64**	**.58**	**.54**	−.04	−.01	**−.12**	**.61**	**.54**	**.56**	**.46**	**.42**	**.36**	**−.18**	**−.15**	**−.18**
Rivalry	.82			**.54**	**.57**	**.57**	**.49**	**.47**	**.29**	**.39**	**.46**	**.42**	**.67**	**.65**	**.61**	**.27**	**.24**	**.17**
Devaluation	.68			**.43**	**.46**	**.45**	**.23**	**.20**	.05	**.20**	**.26**	**.25**	**.53**	**.52**	**.50**	.03	−.01	−.03
Strive for supremacy	.82			**.48**	**.51**	**.50**	**.38**	**.37**	**.20**	**.35**	**.41**	**.37**	**.58**	**.57**	**.53**	**.20**	**.17**	**.11**
Aggressiveness	.72			**.35**	**.37**	**.37**	**.52**	**.53**	**.44**	**.34**	**.40**	**.35**	**.46**	**.42**	**.42**	**.38**	**.38**	**.31**
PNI (*n* = 463)																		
Grandiosity	.91			**.52**	**.60**	**.59**	**.51**	**.49**	**.30**	**.55**	**.61**	**.58**	**.53**	**.56**	**.49**	**.43**	**.35**	**.28**
Exploitativeness	.88			**.67**	**.61**	**.59**	.00	.00	**−.16**	**.51**	**.41**	**.45**	**.55**	**.53**	**.48**	**−.14**	**−.16**	**−.18**
Grandiose fantasy	.87			**.39**	**.50**	**.48**	**.46**	**.43**	**.30**	**.50**	**.61**	**.56**	**.36**	**.38**	**.33**	**.45**	**.37**	**.30**
Self-sacrificing self-enhancement	.83			**.13**	**.23**	**.23**	**.35**	**.36**	**.27**	**.27**	**.33**	**.30**	**.14**	**.22**	**.17**	**.39**	**.34**	**.30**
Entitlement rage	.85			**.29**	**.37**	**.40**	**.67**	**.63**	**.48**	**.29**	**.39**	**.36**	**.49**	**.49**	**.46**	**.53**	**.45**	**.39**
Vulnerability	.94			.07	**.23**	**.28**	**.75**	**.72**	**.62**	.09	**.27**	**.22**	**.34**	**.39**	**.37**	**.71**	**.62**	**.58**
Contigent self-esteem	.93			−.01	**.15**	**.19**	**.72**	**.72**	**.66**	.09	**.28**	**.20**	**.22**	**.27**	**.26**	**.79**	**.73**	**.67**
Hiding the self	.83			*.12*	**.26**	**.30**	**.62**	**.56**	**.46**	.07	**.23**	**.19**	**.36**	**.38**	**.36**	**.52**	**.43**	**.41**
Devaluing	.83			.08	**.20**	**.25**	**.60**	**.57**	**.46**	.07	**.18**	**.17**	**.31**	**.36**	**.32**	**.51**	**.42**	**.40**
HSNS (*n* = 1,285)	.67	**·**	**·**	**.10**	**.15**	**.19**	**.69**	**.67**	**.60**	.05	**.14**	**.07**	**.34**	**.35**	**.35**	**.59**	**.57**	**.53**
DAPP (*n* = 463)	.91	**·**		**.40**	**.49**	**.49**	**.57**	**.55**	**.44**	**.51**	**.63**	**.55**	**.42**	**.41**	**.38**	**.57**	**.50**	**.43**
PES (*n* = 1,027)	.75	**·**	**·**	**.50**	**.51**	**.49**	**.26**	**.26**	**.11**	**.38**	**.42**	**.37**	**.54**	**.51**	**.48**	*.08*	.06	.02
SDT (*n* = 463)																		
Narcissism	.70			**.69**	**.61**	**.57**	−.01	−.03	**−.17**	**.71**	**.63**	**.60**	**.43**	**.37**	**.33**	**−.14**	**−.17**	**−.20**
Machiavellianism	.79			**.50**	**.56**	**.57**	**.42**	**.36**	**.16**	**.28**	**.36**	**.37**	**.64**	**.64**	**.56**	**.18**	.*12*	.07
Psychopathy	.71			**.58**	**.60**	**.59**	**.28**	**.22**	.03	**.28**	**.30**	**.34**	**.71**	**.70**	**.61**	.01	−.06	*−.09*
Personality general																		
NEO-PI-R (*n* = 433)																		
Neuroticism	.94			**−.29**	**−.14**	−.05	**.75**	**.71**	**.67**	**−.23**	−.04	−.05	.05	*.10*	*.10*	**.75**	**.68**	**.64**
Anxiety (N1)	.84			**−.28**	**−.17**	−.07	**.61**	**.59**	**.57**	**−.18**	−.04	−.03	−.03	.01	.02	**.63**	**.59**	**.56**
Angry hostility (N2)	.70			.05	**.14**	**.20**	**.65**	**.55**	**.42**	.02	**.14**	**.15**	**.34**	**.33**	**.28**	**.45**	**.36**	**.31**
Depression (N3)	.89			**−.28**	*−.12*	−.03	**.68**	**.62**	**.58**	**−.25**	−.05	−.04	.04	.09	.09	**.67**	**.59**	**.55**
Self-consciousness (N4)	.77			**−.42**	**−.26**	**−.18**	**.65**	**.67**	**.70**	**−.31**	**−.13**	**−.16**	**−.14**	−.07	−.02	**.78**	**.75**	**.71**
Impulsiveness (N5)	.65			−.01	.03	.03	**.22**	**.25**	**.23**	.00	.05	.06	.09	*.11*	.06	**.23**	**.23**	**.23**
Vulnerability (N6)	.84			**−.33**	**−.20**	**−.13**	**.61**	**.57**	**.57**	**−.30**	**−.13**	**−.15**	−.03	.02	.05	**.63**	**.57**	**.55**
Extraversion	.91			**.42**	**.29**	**.21**	**−.37**	**−.33**	**−.32**	**.57**	**.37**	**.37**	.08	.05	−.01	**−.33**	**−.29**	**−.27**
Warmth (E1)	.77			.04	−.05	*−.10*	**−.36**	**−.25**	**−.17**	**.25**	*.11*	*.12*	**−.26**	**−.23**	**−.25**	**−.18**	*−.12*	*−.10*
Gregariousness (E2)	.80			**.15**	.04	.01	**−.28**	**−.22**	**−.15**	**.26**	**.14**	**.13**	−.07	*−.10*	−.09	**−.16**	*−.12*	−.09
Assertiveness (E3)	.82			**.63**	**.50**	**.45**	**−.27**	**−.32**	**−.36**	**.70**	**.54**	**.54**	**.31**	**.22**	**.20**	**−.36**	**−.38**	**−.37**
Activity (E4)	.69			**.40**	**.30**	**.25**	**−.16**	**−.15**	**−.19**	**.50**	**.35**	**.34**	**.18**	**.14**	.08	**−.19**	**−.17**	**−.17**
Excitement-seeking (E5)	.53			**.44**	**.41**	**.35**	−.04	−.07	**−.17**	**.41**	**.34**	**.33**	**.34**	**.34**	**.25**	**−.16**	**−.18**	**−.19**
Positive emotions (E6)	.86			**.17**	.06	−.02	**−.41**	**−.31**	**−.28**	**.30**	*.12*	**.14**	*−.11*	*−.11*	**−.17**	**−.31**	**−.24**	**−.22**
Openness	.87			.07	.04	.01	**−.20**	**−.14**	**−.15**	**.22**	**.16**	**.18**	*−.12*	*−.12*	**−.16**	*−.10*	−.06	−.09
Fantasy (O1)	.54			*.12*	**.16**	**.15**	.06	.05	.01	**.19**	**.23**	**.25**	.06	.07	.02	.06	.03	.00
Aesthetics (O2)	.79			−.04	−.04	−.05	−.06	−.04	−.01	.07	.04	.06	*−.11*	*−.11*	**−.13**	−.01	.03	.01
Feelings (O3)	.74			−.01	−.05	−.09	−.08	−.01	−.01	**.19**	*.11*	*.10*	**−.15**	**−.14**	**−.22**	.02	.07	.06
Actions (O4)	.69			**.21**	*.11*	.08	**−.32**	**−.27**	**−.27**	**.22**	.09	*.12*	.03	.00	.00	**−.29**	**−.24**	**−.22**
Ideas (O5)	.81			**.15**	**.15**	**.13**	**−.14**	**−.14**	**−.20**	**.23**	**.22**	**.23**	.00	−.01	−.04	*−.12*	**−.13**	**−.16**
Values (O6)	.53			**−.18**	**−.21**	**−.21**	**−.25**	**−.17**	*−.10*	−.05	−.09	−.06	**−.32**	**−.31**	**−.28**	−.05	.01	.00
Agreeableness	.87			**−.68**	**−.69**	**−.70**	**−.32**	**−.19**	.02	**−.42**	**−.46**	**−.46**	**−.82**	**−.74**	**−.69**	.02	**.13**	**.17**
Trust (A1)	.63			*−.11*	**−.20**	**−.28**	**−.57**	**−.45**	**−.25**	.08	−.04	−.04	**−.46**	**−.45**	**−.42**	**−.25**	**−.17**	**−.14**
Straightforwardness (A2)	.64			**−.62**	**−.61**	**−.60**	**−.18**	*−.11*	.01	**−.42**	**−.42**	**−.44**	**−.65**	**−.61**	**−.55**	.02	.08	*.11*
Altruism (A3)	.73			**−.35**	**−.37**	**−.40**	**−.30**	**−.18**	−.09	*−.10*	**−.19**	**−.17**	**−.54**	**−.46**	**−.49**	*−.07*	.00	.03
Compliance (A4)	.67			**−.52**	**−.51**	**−.51**	**−.22**	*−.12*	.09	**−.39**	**−.38**	**−.40**	**−.57**	**−.53**	**−.44**	**.11**	**.19**	**.21**
Modesty (A5)	.75			**−.68**	**−.62**	**−.58**	.02	.07	**.19**	**−.57**	**−.54**	**−.52**	**−.56**	**−.47**	**−.44**	**.18**	**.24**	**.27**
Tender-mindedness (A6)	.69			**−.42**	**−.41**	**−.42**	−.09	−.01	.08	**−.20**	**−.23**	**−.22**	**−.49**	**−.43**	**−.45**	.08	**.15**	**.16**
Conscientiousness	.92			**.18**	.09	.07	**−.22**	**−.17**	**−.20**	**.34**	**.21**	**.16**	−.06	−.08	−.06	**−.17**	**−.14**	**−.13**
Competence (C1)	.69			**.28**	**.16**	**.13**	**−.37**	**−.29**	**−.32**	**.39**	**.23**	**.21**	.00	−.03	−.04	**−.35**	**−.30**	**−.28**
Order (C2)	.71			*.10*	.05	.05	−.06	−.05	−.08	**.23**	**.16**	*.11*	−.04	−.04	−.03	−.02	−.01	−.02
Dutifulness (C3)	.67			−.01	−.08	*−.10*	**−.21**	**−.15**	**−.15**	**.13**	.05	.00	**−.19**	**−.19**	**−.19**	**−.14**	−.09	−.07
Achievement striving (C4)	.70			**.37**	**.28**	**.26**	−.07	−.06	**−.14**	**.54**	**.41**	**.36**	**.13**	*.10*	.09	*−.10*	−.09	*−.11*
Self-discipline (C5)	.87			**.17**	.06	.03	**−.31**	**−.24**	**−.27**	**.26**	**.13**	.08	−.05	−.08	−.07	**−.28**	**−.22**	**−.20**
Deliberation (C6)	.78			−.09	−.08	−.04	.03	.03	.06	.01	.02	.00	**−.14**	**−.14**	−.06	.09	.09	.08
BIS/BAS (*n* = 465)																		
BIS	.84			**−.43**	**−.32**	**−.29**	**.63**	**.65**	**.69**	**−.27**	*−.12*	**−.19**	**−.17**	**−.13**	**−.13**	**.77**	**.76**	**.74**
BAS	.79			**.44**	**.38**	**.34**	.01	.04	−.07	**.52**	**.42**	**.39**	**.28**	**.26**	**.21**	*−.10*	−.09	−.09
AHPS (*n* = 462)																		
Authentic pride	.91			**.39**	**.22**	**.18**	**−.53**	**−.47**	**−.49**	**.43**	**.22**	**.23**	.04	−.02	.00	**−.55**	**−.49**	**−.48**
Hubristic pride	.83			**.43**	**.44**	**.42**	**.16**	**.14**	.06	**.34**	**.38**	**.35**	**.44**	**.40**	**.40**	.07	.02	.00
Personality clinical																		
PID-5 (*n* = 462)																		
Negative affect	.88			**−.25**	*−.10*	−.03	**.66**	**.64**	**.58**	**−.18**	−.02	−.03	.06	**.13**	*.10*	**.64**	**.60**	**.56**
Detachment	.83			−.03	*.11*	*.14*	**.46**	**.34**	**.26**	**−.22**	−.02	−.05	**.29**	**.29**	**.29**	**.28**	**.19**	**.17**
Psychoticism	.86			**.16**	**.26**	**.29**	**.41**	**.37**	**.21**	.03	**.15**	**.15**	**.38**	**.41**	**.36**	**.21**	**.15**	**.13**
Antagonism	.86			**.72**	**.72**	**.71**	**.20**	**.14**	−.05	**.48**	**.51**	**.48**	**.77**	**.72**	**.71**	−.02	−.09	**−.13**
Disinhibition	.83			.07	**.18**	**.23**	**.49**	**.48**	**.40**	−.07	.08	*.11*	**.35**	**.39**	**.35**	**.39**	**.35**	**.32**
MSI-BPD (*n* = 463)	.83	**·**		.04	**.16**	**.21**	**.53**	**.44**	**.37**	−.02	*.11*	*.11*	**.30**	**.33**	**.32**	**.38**	**.29**	**.27**
IPO (*n* = 275)	.77		**·**	**.43**	**.46**	**.47**	**.55**	**.49**	**.37**	**.33**	**.35**	**.35**	**.51**	**.53**	**.50**	**.19**	**.16**	*.14*
Identity diffusion	.65			.07	*.13*	*.13*	**.65**	**.63**	**.58**	**.20**	**.23**	**.20**	*.13*	**.17**	**.16**	**.56**	**.53**	**.49**
Primitive defenses	.70			**.50**	**.51**	**.54**	**.41**	**.37**	**.19**	**.34**	**.35**	**.40**	**.57**	**.58**	**.55**	.00	−.02	−.03
Reality testing	.72			**.43**	**.43**	**.42**	**.20**	*.14*	.08	**.23**	**.23**	**.22**	**.48**	**.47**	**.46**	−.11	*−.14*	*−.12*
Interpersonal																		
IIP-32 (*n* = 465)	.82	**·**		**−.18**	−.03	−.02	**.61**	**.57**	**.55**	**−.21**	−.02	−.06	**.12**	**.16**	**.14**	**.58**	**.54**	**.51**
Domineering (PA)	.65			**.44**	**.49**	**.45**	**.43**	**.38**	**.23**	**.33**	**.39**	**.34**	**.57**	**.55**	**.48**	**.19**	**.17**	*.11*
Intrusive (NO)	.63			**.18**	**.23**	**.21**	**.33**	**.35**	**.24**	**.23**	**.28**	**.26**	**.23**	**.25**	**.18**	**.27**	**.26**	**.19**
Overly nurturant (LM)	.64			**−.26**	**−.19**	**−.16**	**.21**	**.24**	**.26**	*−.11*	**−.06**	−.04	**−.21**	**−.13**	**−.15**	**.28**	**.27**	**.27**
Exploitable (JK)	.66			**−.44**	**−.36**	**−.33**	**.21**	**.23**	**.34**	**−.31**	**−.21**	**−.19**	**−.35**	**−.30**	**−.28**	**.41**	**.40**	**.40**
Nonassertive (HI)	.72			**−.48**	**−.38**	**−.34**	**.29**	**.31**	**.41**	**−.43**	**−.29**	**−.30**	**−.30**	**−.25**	**−.21**	**.45**	**.44**	**.46**
Socially avoidant (FG)	.75			**−.31**	**−.19**	**−.18**	**.45**	**.42**	**.44**	**−.38**	**−.18**	**−.23**	−.02	−.01	.00	**.48**	**.45**	**.44**
Cold (DE)	.80			−.01	.07	.08	**.34**	**.26**	**.23**	**−.16**	−.05	−.09	**.23**	**.24**	**.23**	**.21**	**.18**	**.18**
Vindictive (BC)	.63			**.35**	**.39**	**.36**	**.37**	**.28**	**.16**	*.11*	**.22**	**.16**	**.55**	**.49**	**.47**	**.16**	**.13**	.07
BPAQ (*n* = 465)	.74	**·**		**.20**	**.27**	**.26**	**.59**	**.54**	**.38**	.06	**.14**	**.13**	**.48**	**.49**	**.39**	**.31**	**.28**	**.24**
Anger	.75			.06	*.11*	*.09*	**.55**	**.51**	**.42**	.05	*.12*	.08	**.28**	**.27**	**.19**	**.37**	**.35**	**.31**
Hostility	.69			*−.12*	.02	.07	**.69**	**.65**	**.55**	**−.18**	−.02	−.05	**.22**	**.28**	**.26**	**.58**	**.51**	**.49**
Physical aggression	.80			**.24**	**.25**	**.24**	**.17**	**.14**	.06	.06	.05	.07	**.37**	**.37**	**.32**	−.04	−.05	−.05
Verbal aggression	.55			**.38**	**.34**	**.30**	.02	−.01	**−.15**	**.29**	**.22**	**.26**	**.36**	**.34**	**.24**	**−.21**	**−.20**	**−.22**
ECR-RD12 (*n* = 1,029)	.72	**·**	**·**	.04	**.10**	**.11**	**.46**	**.43**	**.39**	.03	**.10**	.04	**.20**	**.22**	**.22**	**.38**	**.36**	**.34**
Anxiety	.81			−.01	.04	*.07*	**.49**	**.48**	**.41**	.03	**.08**	.04	**.14**	**.17**	**.15**	**.43**	**.41**	**.39**
Avoidance	.50			**.14**	**.17**	**.16**	**.31**	**.28**	**.24**	*.06*	**.12**	*.07*	**.27**	**.26**	**.26**	**.22**	**.20**	**.19**
LAS (*n* = 383)																		
Agape	.89			*−.11*	−.07	−.08	.08	.08	*.10*	−.06	−.06	−.01	−.08	−.04	−.10	.09	*.11*	*.11*
Eros	.91			.02	−.02	−.03	**−.14**	*−.12*	−.08	.05	.00	.05	−.06	−.07	−.10	**−.13**	−.10	−.08
Ludus	.81			**.36**	**.38**	**.33**	**.17**	**.15**	.04	**.27**	**.29**	**.24**	**.39**	**.38**	**.32**	.05	.02	−.01
Mania	.84			−.07	.02	.00	**.47**	**.48**	**.42**	−.03	.09	.03	*.12*	**.13**	.07	**.45**	**.44**	**.40**
Pragma	.86			**.22**	**.24**	**.26**	**.20**	**.15**	**.15**	**.25**	**.28**	**.26**	**.19**	**.17**	**.22**	**.14**	.09	.08
Storge	.82			−.08	−.07	−.04	.02	.02	.07	−.04	−.06	−.01	−.08	−.06	−.04	.03	.03	.05
Psychological adjustment																		
RSES (*n* = 2,387)	.92	**·**	**·**	**.26**	**.13**	**.07**	**−.61**	**−.55**	**−.54**	**.29**	**.12**	**.14**	*−.05*	**−.10**	**−.11**	**−.61**	**−.55**	**−.52**
SWLS (*n* = 462)	.88	**·**		**.22**	.05	.01	**−.51**	**−.42**	**−.41**	**.28**	.08	.08	*−.10*	**−.15**	**−.14**	**−.46**	**−.37**	**−.38**
Psychological maladjustment																		
BSI Global Severity Index (*n* = 466)	.96	**·**	**·**	.01	.04	.09	**.52**	**.47**	**.42**	−.01	.02	−.01	**.17**	**.19**	**.22**	**.42**	**.37**	**.34**
CES-D (*n* = 462)	.90	**·**		**−.25**	*−.10*	*−.06*	**.60**	**.55**	**.49**	**−.28**	*−.11*	*−.12*	*.10*	**.15**	*.10*	**.54**	**.50**	**.47**

*Note*. Correlations significant at *p* < .05 level are printed in italic, correlations significant at *p* < .01 are printed in bold. Correlations for the FFNI-LF and -SF are displayed jointly (preceding columns indicate which version[s] coefficients are based upon) so that the coefficients of the LF/SF can be compared to those of the BF and SSF within the same individuals.^a^ FFNI = Five-Factor Narcissism Inventory; LF = long form; SF = short form; BF = brief form; SSF = super-short form; NPI = Narcissistic Personality Inventory; NARQ = Narcissistic Admiration and Rivalry Questionnaire; PNI = Pathological Narcissism Inventory; HSNS = Hypersensitive Narcissism Scale; DAPP = Dimensional Assessment of Psychopathology; PES = Psychological Entitlement Scale; SDT = Short Dark Triad; NEO—PI—*R* = NEO—Personality Inventory—Revised; BIS/BAS = Behavioral Inhibition/Behavioral Activation Scales; AHPS = Authentic Hubristic Pride Scale; PID-5 = Personality Inventory for *DSM-5*; MSI-BPD = McLean Screening Instrument for Borderline Personality Disorder; IPO = Inventory of Personality Organization; IIP-32 = Inventory of Interpersonal Problems-32; BPAQ = Buss-Perry Aggression Questionnaire; ECR-RD12 = Experience in Close Relationships-Revised Questionnaire; LAS = Love Attitudes Scale; RSES = Rosenberg Self-Esteem Scale; SWLS = Satisfaction with Life Scale; BSI = Brief Symptom Inventory; CES-D = Center for Epidemiologic Studies Depression Scale.

a We note that validity coefficients for the FFNI-LF and -SF were generally highly similar, and comparability of the two questionnaire versions has been established previously (Sherman et al., 2015).

##### Narcissism and Related Constructs

As hypothesized, FFNI grandiose narcissism displayed generally high and consistent correlations with other measures of grandiose narcissism (NPI, NARQ), and—to a smaller extent—the PNI grandiose scales. Correlations with vulnerable narcissism measures (HSNS, PNI vulnerable) were low to moderate. FFNI vulnerable narcissism, on the contrary, was not generally associated with those narcissism scales assessing primarily agentic aspects of grandiose narcissism (NPI leadership/authority and grandiose exhibitionism, NARQ admiration), but was associated with antagonistic scales (NPI entitlement/exploitativeness and NARQ rivalry, though to a lesser extent in the SSF). FFNI vulnerable narcissism was strongly and consistently associated with other measures of vulnerable narcissism. Both, FFNI grandiose and vulnerable narcissism were related to the DAPP as a clinical narcissism scale (though vulnerable narcissism somewhat stronger), and both were associated with psychological entitlement, as predicted by theory (however, the vulnerable score of the SSF displayed a low association). Within the three-factor model, as expected, FFNI agentic narcissism was primarily and strongly associated with other agentic narcissism scales, but also antagonistic narcissism scales, and displayed low-to-moderate associations with measures of vulnerable narcissism. FFNI antagonistic narcissism was primarily associated with other antagonistic narcissism scales, though also with agentic narcissism scales, substantially associated with vulnerable narcissism scales, and psychological entitlement. FFNI neurotic narcissism was slightly negatively associated with agentic narcissism scales, partially associated with antagonistic scales (primarily NARQ rivalry), and strongly associated with vulnerable narcissism scales (PNI vulnerable, HSNS). Associations with psychological entitlement were low to negligible. Regarding the Dark Triad traits, as assessed with the SDT, FFNI grandiose narcissism, particularly its agentic aspects, displayed the strongest convergent associations with SDT narcissism (though also being substantially related to Machiavellianism and psychopathy), whereas antagonistic aspects were more strongly related to SDT Machiavellianism and psychopathy.

##### General Personality Measures

In the NEO-PI-R as a general FFM personality measure, FFNI grandiose narcissism reflected—as expected—primarily in an extraverted and disagreeable personality profile. We also observed negative correlations with facets of neuroticism as well as sporadic associations with openness and conscientiousness (particularly achievement striving). Vulnerable narcissism—as expected—reflected primarily neuroticism and disagreeableness (though the latter was not as pronounced in the SSF). Vulnerable narcissism was further considerably associated with introversion and low conscientiousness. Within the three-factor model, it is interesting to note that though FFNI agentic narcissism did show the expected associations with NEO extraversion; relations with disagreeableness were also prominent. We further observed substantial positive associations with openness and conscientiousness. FFNI antagonistic narcissism was mainly and consistently associated with disagreeableness (though also with some facets of extraversion), and FFNI neurotic narcissism was strongly and consistently associated with NEO neuroticism, and also considerably associated with introversion. Interestingly, FFNI neurotic narcissism was slightly positively associated with some aspects of agreeableness.

Consistent with predictions, grandiose narcissism—particularly its agentic aspects—was associated with behavioral activation in the BIS/BAS, whereas vulnerable narcissism—particularly its neurotic aspects—was associated with behavioral inhibition. Grandiose narcissism was related mainly to hubristic but also authentic pride. Closer differentiation in the three-factor model shows that associations with hubristic pride is mostly due to antagonistic narcissism. Vulnerable narcissism—particularly neurotic narcissism—was negatively associated with authentic pride.

##### Clinical Personality Measures

Within the clinical FFM dimensions as assessed by the PID-5, FFNI grandiose narcissism was mainly associated with antagonism. Interestingly, also psychoticism and disinhibition displayed positive associations. Vulnerable narcissism, as expected, displayed the strongest associations with negative affect, but correlated with all of the PID-5 dimensions (least with antagonism, which was insignificant in the SSF). Inspection within the three-factor model shows that it is particularly FFNI antagonistic narcissism which shows associations to all PID-5.

FFNI antagonistic narcissism and vulnerable narcissism displayed moderate correlations with a *DSM*-based measure of borderline personality disorder (MSI-BPD), which also reflects in substantial correlations of borderline personality disorder and vulnerable and, to some extent, grandiose narcissism. All FFNI narcissism dimensions displayed substantial associations with the IPO as a measure of general personality functioning. Antagonistic narcissism was more related to primitive defenses and impaired reality testing, whereas neurotic narcissism was more related to identity diffusion.

##### Interpersonal Experience and Behavior

In the interpersonal circumplex, grandiose narcissism was mainly related to interpersonal problems related to domineering (PA) and also vindictive (BC) interpersonal styles. Both were related more to the antagonistic than the agentic aspects. Vulnerable narcissism was associated with a wide array of interpersonal problems (as also evident in high overall correlations), most strongly those related to a socially avoidant interpersonal style. This was attributable to neurotic rather than antagonistic aspects of narcissism. Relations with interpersonal problems were generally highly similar for the different FFNI versions.

Both grandiose and vulnerable narcissism were related to aggression, and, as expected, the associations were most pronounced for antagonistic narcissism within the three-factor model (though also neurotic narcissism was related to aggression). Grandiose narcissism was weakly related to attachment avoidance, whereas vulnerable narcissism was substantially related to both, attachment anxiety and avoidance. Within the three-factor model, it was mostly neurotic, but also antagonistic narcissism, which related to both dimensions. Finally, grandiose narcissism was related to game-playing (ludus) and also pragmatic (pragma) love styles, and the same pattern was evident for its constituent three-factor dimensions agentic and antagonistic narcissism. Vulnerable narcissism and its neurotic aspects were further associated with an obsessive love style (mania).

##### Psychological Adjustment and Maladjustment

As expected, grandiose narcissism was moderately positively associated with self-esteem and life satisfaction (though this latter correlation was not evident in the SSF, and also the correlation with self-esteem was low in the SSF), whereas vulnerable narcissism was negatively associated with both. Correlations within the three-factor model show that positive associations with adaptive adjustment are due to agentic narcissism, whereas negative associations are due primarily due to neurotic, but also antagonistic narcissism.

Grandiose narcissism largely displayed zero-associations with psychological maladjustment (though the LF was negatively related to depression), whereas vulnerable narcissism showed strong positive associations with symptom load and depression. Within the three-factor model, agentic aspects of narcissism were not associated with symptom load and negatively associated with depression (though, again, only in the LF), antagonistic aspects were slightly positively associated with both, and neurotic aspects displayed strong and consistent associations.

##### Differences Relating to Clinical Criteria and Groups

To investigate the validity of the German FFNI regarding more objective clinical and forensic criteria, we studied differences relating to prior/current mental disorder diagnoses (any diagnosis, see methods) and status as incarcerated young criminal offender. Table 5 shows FFNI scale differences as a function of these. In line with our expectations, those who reported diagnoses had significantly higher FFNI scores on vulnerable narcissism, its neurotic aspects, and all facets of neurotic narcissism (*shame*, *indifference* [reversed], and *need for admiration*), as well the antagonistic narcissism facet *distrust*. They had lower scores on the antagonistic narcissism facet *lack of empathy*. This pattern of results was the same for all questionnaire versions.

**Table 5.** Differences in FFNI Scales Related to Mental Disorder Diagnoses (Samples 6 and 7) and Incarceration Status (Sample 8).

	Mental disorder diagnosis						Incarcerated criminal offender					
	No diagnosis (*N* = 363)			Prior/current diagnosis (*N* = 103)			No (*N* = 2,853)			Yes (*N* = 68)		
	SF	BF	SSF	SF	BF	SSF	SF	BF	SSF	SF	BF	SSF
Two-factor model												
Grandiose Narcissism	2.25	2.21	2.23	2.16	2.12	2.18	2.34	2.22	2.25	2.78**	2.75**	2.79**
Vulnerable Narcissism	2.80	2.79	2.51	3.14**	3.13**	3.00**	2.85	2.98	2.60	2.89	2.79**	2.35**
Three-factor model												
Agentic Narcissism	2.72	2.61	2.68	2.67	2.57	2.65	2.83	2.69	2.78	2.86	2.75	2.83
Antagonism Narcissism	2.07	2.08	1.92	2.05	2.05	1.91	2.18	2.14	1.91	2.77**	2.78**	2.61**
Neurotic Narcissism	3.28	3.23	3.20	3.69**	3.65**	3.70**	3.24	3.34	3.38	2.65**	2.49**	2.43**
Facets												
Reactive anger	2.42	2.57	—	2.51	2.62	—	2.60	2.90	—	3.01**	3.12^†^	—
Shame	3.22	2.95	—	3.68**	3.50**	—	3.24	3.01	—	2.63**	2.41**	—
Indifference	2.31	2.32	—	2.09*	2.07*	—	2.32	2.22	—	3.29**	3.35**	—
Need for admiration	2.93	3.06	—	3.48**	3.50**	—	2.81	3.22	—	2.61*	2.40**	—
Exhibitionism	2.92	2.83	—	2.83	2.71	—	2.92	3.00	—	3.00	2.81	—
Authorativeness	2.81	2.67	—	2.68	2.58	—	2.98	2.86	—	2.79^†^	2.76	—
Grandiose fantasies	2.20	2.29	—	2.23	2.34	—	2.44	2.23	—	2.75**	2.66**	—
Manipulativeness	2.40	2.50	—	2.44	2.54	—	2.45	2.56	—	2.78*	2.82^†^	—
Exploitiveness	1.65	1.72	—	1.57	1.64	—	1.70	1.71	—	2.24**	2.29**	—
Entitlement	1.68	1.59	—	1.58	1.46	—	1.85	1.58	—	2.23**	2.22**	—
Lack of empathy	1.82	1.76	—	1.63**	1.53**	—	1.73	1.57	—	2.71**	2.65**	—
Arrogance	1.92	1.81	—	1.83	1.78	—	2.10	1.81	—	2.86**	2.80**	—
Acclaim seeking	2.94	2.67	—	2.92	2.65	—	3.00	2.69	—	2.92	2.75	—
Thrill seeking	2.06	2.10	—	1.94	1.97	—	2.25	2.21	—	3.04**	3.11**	—
Distrust	2.62	2.60	—	2.89*	2.87**	—	2.74	2.77	—	3.31**	3.24**	—

*Note*. FFNI = Five-Factor Narcissism Inventory; SF = long form; BF = brief form; SSF = super-short form.

* and ** indicate significant differences to the reference group at *p* < .05 and *p* < .01, respectively, ^†^indicates trends at *p* < .10, as assessed by independent samples *t*-tests (corrected for unequal variance, if applicable).

Also as expected, the sample of young offenders had significantly higher scores on grandiose narcissism and its constituent factor antagonistic narcissism, as well as significantly lower scores on neurotic narcissism in all questionnaire versions. Lower scores on overall vulnerable narcissism were evident only in the SSF and the Brief From (presented in the following). We observed significant differences on nearly all facets (with similar effect patterns for different questionnaire versions; *authoritativeness* facet by trend only in the SF), which point in the direction of a highly grandiose-antagonistic narcissism profile, with low scores on neurotic aspects (*need for admiration* and *shame*).

### Conclusion

Study 1 showed that the German FFNI is a reliable and valid measure of narcissism within both contemporary structural models, namely the two- and three-factor models of narcissism. Tests of its factorial structure showed that both models fit the data when an adequate level of complexity is taken into account. However, shorter versions of the FFNI better capture the intended structure of the FFNI at facet- and factor-level than the FFNI-LF, which displayed poor fit unless including cross-loadings, and partly insignificant facet loadings when cross-loadings were considered. These analyses underpin the necessity of direct confirmatory tests of the internal structure of the different FFNI versions, which have rarely been performed (for FFNI-SF, see Papageorgiou et al., 2021).

Regarding validity, the FFNI factors displayed high convergent validity with other narcissism measures. Correlation patterns with criterion validity measures were generally similar to those of previous studies and show that FFNI grandiose narcissism entails both adaptive and maladaptive aspects, which relate more strongly to agentic and antagonistic narcissism, respectively. Vulnerable narcissism and both of its constituent factors are characterized mainly by personality dysfunction.

Although findings from Study 1 speak to the general validity of the FFNI, they also point to some problems in the factorial structure in the FFNI-LF. In study 2, we aim to construct a brief from of the FFNI which optimizes both its internal structure and external validity.

## Study 2: Brief Form Construction and Validation

### Aims

While Study 1 showed that the German FFNI generally is a reliable and valid measure of narcissism within both the two- and three-factor models, and factor- as well as facet-level scores have validity for different criteria, there are also ways in which the FFNI could be further developed. First, the FFNI-LF is not only time-consuming to administer (148 items), which may impede its use in certain contexts, but also displays problems in its structure, in the way that the item set might not be optimally suited to capture the facet and factor structure in an unidimensional manner. Second, available short forms—the 60-item FFNI-SF and the 15-item FFNI-SSF—have been constructed selecting items based on high Item Response Theory discrimination parameters (SF; Sherman et al., 2015) or principal component main loadings (SSF; Packer West et al., 2021). These approaches both optimize the internal consistency of the scale, but not other relevant criteria such as model fit and factor-level correlations with other narcissism measures. Because of the focus on high main loadings, the existing 60-item version also contains semantically redundant items that maximize this criterion (see FFNI brief form (FFNI-BF) construction section). The aim of Study 2 is therefore to select a brief form that would achieve sufficient model fit for the three-factor structure, as well as reliability (i.e., factor saturation McDonald’s ω), and validity with respect to established measures of agentic, antagonistic, and neurotic narcissism.

We used the item selection procedure ACO to construct a 30-item brief form of the FFNI which preserves its structure and maximizes model fit, reliability, and validity of the three factors. We then compare the different FFNI versions regarding their internal structure and validity (self-report measures and more objective clinical/forensic criteria, as in Study 1).

### Method

#### Samples, Measures, and Analysis Plan

We used the samples and measures described in Study 1 for the ACO item selection, cross-validation of the solution, and further factorial structure and criterion validity tests of the brief form. Specifically, for the item selection process (see below), we used data from those samples in which the FFNI-LF was administered (samples 1-4, *N* = 1,823, see Table 1). We then cross-validated the solution using data from the samples in which the FFNI-SF was administered (samples 5-8, *N* = 1,098, see Table 1). Finally, we pooled all samples (*N* = 2,921) for further tests of the factorial structure in which we also evaluated the models described in Study 1, and examined criterion validity.

#### FFNI Brief Form (FFNI-BF) Construction

We constructed a 30-item brief form of the FFNI using ACO (Olaru et al., 2019; Schroeders et al., 2016). ACO is a metaheuristic algorithm that selects and evaluates item sets based on (combinatorial) scale-level properties such as model fit or reliability. Traditional short scale development procedures generally rely on item-level information derived in the full scale (e.g., factor loadings of the 60-item FFNI-SF), even though the criteria of interest can only be derived for combinations of items (e.g., reliability). In addition, selecting items based on properties in the initial full scale relies on the assumption that these do not change as soon as items are removed (e.g., initial factor loadings corresponding to the short scale loadings). ACO selects and evaluates combinations of items and learns over the course of several iterations which items are particularly well suited to optimize the given criteria (e.g., model fit). Because it selects items based on scale-level properties of the final instead of the initial scale, it is superior to traditional item selection techniques (for more details, see Olaru et al., 2015, 2019).

Here, we used ACO to select a 30-item brief form with two items per facet. As the measurement model for which ACO selected the items, in keeping with the FFNI idea of narcissistic variants of FFM traits (Glover et al., 2012), we specified a three-factor model with agentic, antagonistic, and neurotic narcissism as factors encompassing 16, 8, and 6 items (i.e., two items per facet; see method section). To account for the facet structure of the scale (i.e., items from a common facet correlating higher with each other), we added residual correlations between item-pairs from a common facet. This is equivalent to specifying nested orthogonal facet factors in a bifactor model. We chose this approach over a higher-order model for the ACO item selection because higher-order models with only two indicators per first-order factor caused convergence problems and yielded a large number of negative residual variances in initial ACO runs. The used model is more robust and considers variance at the factor and facet level, thus capturing the intended factor- and facet structure of the FFNI.

Based on this model, we optimized (1) the model fit, more specifically RMSEA, CFI, and SRMR, (2) the reliability (factor saturation McDonald’s ω) of the three factors, and (3) their correlations with external criteria. Regarding the latter, we maximized the correlations between the FFNI factors of agentic narcissism with NPI leadership/authority as well as NARQ admiration, antagonistic narcissism with NPI entitlement/exhibitionism, NARQ rivalry, and the PES, and neurotic narcissism with the HSNS and PNI vulnerable dimension.^6^ Each criterion was logit transformed to a scale of 0 to 1 (for an illustration, see Olaru et al., 2019), and the average across the three criteria was used as the overall optimization criterion. The exact optimization function can be found in the R script in the OSF (https://osf.io/ae7vr/).

As item selection pool, we used the FFNI-SF (Sherman et al., 2015). We chose the short form as a starting point to ensure that the current scale was nested in the FFNI-SF, and also because this would give us the largest sample size to work with (see above). From the initial 60-item pool, we removed six highly content redundant items^7^ to ensure that pairs of semantically similar items were not selected to increase the internal consistency of the scale at the cost of construct coverage. We further excluded two reverse-coded items^8^ from the initial pool, as those would otherwise have been the only reverse-coded items in the pool, leading to a 52-item selection pool.

We used ACO with a comparison of 50 models per iteration (i.e., “ants”) and a stopping criterion of 30 iterations without improvement to the previously best solution. Because ACO is a probabilistic algorithm, a single run may not be sufficient to find an optimal solution. We thus ran it 10 times with different random generator seeds and used the best solution of the ten runs as final solution. To ensure that the solution was robust and generalizable across samples, we used cross-validation. Specifically, we selected the brief form solely in the combined sample in which participants filled in the long form (selection sample; *N* = 1,823). To evaluate the robustness of the model fit, reliability and correlations of the scale, we then estimated these values for the final brief form on the combined sample in which participants filled in the short form (validation sample; *N* = 1,098). Because these samples had been collected independently from each other in the context of different studies, this approach should yield a good indication of the generalizability of the selected scale properties. Finally, for consistency with the analyses presented in Study 1, we re-evaluated the factorial structure in the overall pooled sample within the models presented there.

### Results and Discussion

#### ACO Item Selection

The 30 items selected by ACO for the Brief Form can be found in Supplemental Table S1 and in the OSF (https://osf.io/ae7vr/). The BF showed largely satisfactory CFA model fit on the selection sample (RMSEA = .069; CFI = .900; SRMR = .072), as well as in the validation sample (RMSEA = .073; CFI = .904; SRMR = .075), and also in the pooled sample (see next section). Factor saturation omega was on average *ω* = .76 in the selection sample (agentic narcissism *ω* = .75; antagonistic narcissism *ω* = .75; neurotic narcissism *ω* = .79), and *ω* = .77 for all three factors in the validation sample. The average correlation between the three factors and the related scales used in the optimization was comparable across the selection and validation scale (selection: *r* = .58; validation: *r* = .61; for single correlations see Table 4). The three brief form scale scores correlated on average *r* = .92 with the scores of the original 60-item short form (agentic narcissism *r* = .94; antagonistic narcissism *r* = .92; neurotic narcissism *r* = .92). In summary, the 30-item brief form yielded satisfactory psychometric properties, which were robust across different samples, and was highly correlated with the 60-item version, thus maintaining construct coverage.

#### Psychometric Properties of the FFNI-BF

The 30-item FFNI-BF displayed good internal consistencies ranging from .72 ≤ α ≤ .84 at factor-level (two- and three-factor models) and from .60 ≤ α ≤ .88 at facet level. Internal consistencies were generally in between those of the FFNI-SF and FFNI-SSF, as it can be expected given the different scale lengths. The pattern of intercorrelations among the factors of the two- and three-factor models was generally highly similar to those of other scale versions (see Table 2).

To re-evaluate the factor structure of the FFNI-BF within the hierarchical CFA, ESEM, and ESEM-within-CFA framework, we tested the same models^9^ as described in Study 1 in the overall pooled sample. As Table 3 shows, similar to the long form, we observed generally better fit for models which consider the nested structure, and which consider cross-loadings of items at the facet level. The FFNI-BF showed largely acceptable and—according to most indices—superior fit than other scale versions in higher-order CFA models, in particular for the three-factor model for which the items were selected (model 4, two-factor: RMSEA = .070, CFI = .814, SRMR = .102; model 7, three-factor: RMSEA = .054, CFI = .892, SRMR = .072). For consistency reasons we also estimated the FFNI-BF in ESEM-within-CFA models (model 11, two-factor: RMSEA = .051, CFI = .955, SRMR = .037; model 14, three-factor: RMSEA = .040, CFI = .973, SRMR = .026). We note, however, that ESEM-within-CFA models, despite their good fit, also yielded parameter estimates which were out of bounds. This likely results from over-fitting a model whose loadings have already been optimized (leading to collinearity when further residual variance is accounted for by the model), in combination with the small number of indicators per facet. The use of ESEM thus seems neither appropriate nor necessary for the FFNI-BF, given the model used for the construction thereof has already been optimized. We recommend the use of more parsimonious traditional hierarchical CFA models or correlated factor model with residual correlations between items of the same facet (selection model used) in future studies. We further note that although fit was better for the optimized three-factor solution than for two-factor models, fit of the two-factor models was similar to the FFNI-SF, and superior to the FFNI-LF. Supplemental Table S1 displays loadings of the FFNI-BF (hierarchical CFA three-factor model).

#### Criterion Validity of the FFNI-BF

The FFNI-BF displayed generally highly similar validity as did longer scale versions regarding measures of narcissism and related constructs, general and clinical personality measures, measures of interpersonal functioning, as well as adaptive and maladaptive psychological adjustment (see Table 4). We observed strong convergence between the FFNI-BF grandiose narcissism factor and other measures of grandiose narcissism (NPI, NARQ) and, to a smaller extent, the PNI grandiosity scales (as in the longer versions). Grandiose narcissism was also correlated with psychological entitlement and composite measures of narcissism (DAPP, SDT narcissism) to a similar extent as in other scale versions. FFNI-BF vulnerable narcissism was correlated strongly to other vulnerable narcissism scales (HSNS and PNI vulnerability) and was also correlated to psychological entitlement and composite measures of narcissism. Of note, the correlation between vulnerable narcissism and psychological entitlement (PES) was within the expected range in the FFNI-BF (*r* = .26) while being weak in the FFNI-SSF (*r* = .11), and similar differences were evident for the entitlement/exploitativeness factor of the NPI, the entitlement rage facet of the PNI, and more generally the disagreeableness/antagonism measures (NEO-PI-R and PID-5). This is likely a result of including the correlation with entitlement—a core characteristic of both forms of narcissism (Krizan & Herlache, 2018)—as an optimization criterion. Within the three-factor model, we also observed strong convergent validity of the FFNI-BF agentic, antagonistic, and neurotic narcissism factors with other measures of agentic, antagonistic, and neurotic narcissism. Specifically, agentic narcissism correlated most strongly with NPI leadership/authority and NARQ admiration, whereas antagonistic narcissism correlated most strongly with NPI entitlement/exploitativeness and NARQ rivalry. Both displayed substantial associations with composite measures of narcissism and psychological entitlement (PES), the latter being more pronounced for antagonistic than for agentic narcissism, as theoretically intended (Weiss et al., 2019). FFNI-BF neurotic narcissism, as in the other scale versions, displayed the highest correlations with PNI vulnerability (though also substantially related to PNI grandiosity), was unrelated to psychological entitlement (PES), and slightly negatively related to agentic measures of narcissism, which has implications for the interpretation of this factor (see general discussion).

Correlations of the FFNI-BF with other personality measures and further external validity criteria were generally highly similar to those of the other scale versions reported in Study 1, with some notable exceptions: while agentic narcissism correlates most strongly with aspects of extraversion related to social dominance (NEO-PI-R assertiveness), the longer versions also display a substantial correlations with aspects related to positive emotionality (NEO-PI-R positive emotions, *r* = .30), which is only weakly the case in the novel BF (*r* = .12) or the recently developed SSF (*r* = .14). Similarly, agentic narcissism is less strongly related to authentic pride in the shorter versions (*r* = .43 vs. *r* = .22 / *r* = .23), and less strongly related to self-esteem (*r* = .29 vs. *r* = .12 / *r* = .14) as well as life satisfaction (*r* = .28 vs. *r* = .08 / *r* = .08). Taken together, this shows that shorter scales draw less strongly upon adaptive aspects of extraversion, which potentially make them more suitable measures of agentic-extraverted narcissism, rather than extraversion per se (see general discussion).

Finally, the novel BF (and also the SSF) displayed largely the same pattern of mean differences between groups with and without mental disorders diagnoses or incarceration status (see Table 5). Diagnoses were related to higher scores on vulnerable narcissism and its constituent factor neurotic narcissism (but not the other factors) and all of its facets, as well as the antagonistic narcissism facet *distrust*, and lower scores on *lack of empathy*. Offenders had higher scores on grandiose narcissism and its constituent factor antagonistic narcissism (but not agentic narcissism) and lower scores on vulnerable narcissism (note that this was not the case in longer versions) and its constituent factor neurotic narcissism (in all versions). At facet level, incarceration status was related to higher scores in the facets *reactive anger* (though only by trend), *indifference*, *grandiose fantasies*, *manipulativeness* (by trend), *exploitativeness*, *entitlement*, *lack of empathy*, *arrogance*, *thrill seeking*, and *distrust*, and lower scores in *shame* and *need for admiration*. The trend-level difference observed for *authoritativeness* in Study 1 was not evident in the BF. Taken together, the FFNI-BF is well suited to capture different characteristic FFNI profiles (see Study 1) at factor and facet level.

### Conclusion

Study 2 presented the construction and validation of a 30-item brief form of the FFNI using the metaheuristic ACO algorithm. Results show that the FFNI-BF is a reliable and valid measure with high correlations to the FFNI-SF (which served as the basis for item selection; .92 ≤ r ≤ .94), an optimized internal structure (based on non-content-redundant items) resulting in superior fit in confirmatory analyses (without the use of ESEM), and high validity with respect to a range of relevant self-report variables and more objective clinical and forensic criteria. Specifically, we found highly similar associations with external criteria as for longer scale versions using a smaller number of items, making the FFNI-BF a highly efficient measure of narcissism within both the two- and three-factor model. One particular advantage compared to the recently developed SSF might be that the vulnerable narcissism factor in the BF does capture entitlement, which is regarded as a core characteristic of narcissism. Also, the FFNI-BF allows for facet-level scoring and yields similar facet-level differences with respect to clinical and forensic criteria as does the FFNI-SF. Taken together, the FFNI-BF may be recommended as a concise measure of narcissism when scores at the two- or three-factor level are needed, and may be interpreted also at the facet level in group-level statistics.

## General Discussion

The main aims of the studies presented here were to validate a German version of the FFNI—a measure covering the spectrum of narcissism from grandiose to vulnerable including agentic, antagonistic, and neurotic aspects from an FFM perspective—and to construct a brief form optimizing its internal structure and external validity. Overall, the data provide robust evidence for the validity of the German FFNI, including the newly devised 30-item FFNI-BF.

### Internal Consistencies and Factor Structure

The two and three-factor model scores of the German FFNI displayed high internal consistencies across all scale versions (LF and SF: .89 ≤ α ≤ .95, BF: 72 ≤ α ≤ .84, SSF: .60 ≤ α ≤ .73). At facet level, coefficients were between .70 ≤ α ≤ .89 for the LF and SF, and .60 ≤ α ≤ .88 in the new BF. These are similar to the original English scales (Glover et al., 2012; SF: .73 ≤ α ≤ .93; Sherman et al., 2015).

Confirmatory factor analyses showed generally acceptable model fit for most FFNI versions when an adequate level of complexity was taken into account: fit was better for hierarchical than nonhierarchical models, which shows that considering uniqueness at item and facet level is necessary when aligning FFNI data with the intended factor structure. When modeled only at facet level, fit was comparable to a previous study involving a confirmatory test of the FFNI-SF (Papageorgiou et al., 2021). Model fit of the FFNI-LF, -SF, and -SSF was better for ESEM models considering cross-loadings than for traditional CFA models. However, such models might not be feasible for researchers or practitioners in each case (i.e., cross-loadings are not commonly considered when scoring a questionnaire). Also, we note that the FFNI-LF, in contrast to other scale versions, did not generally show acceptable fit unless using ESEM, and exhibited problems in its factorial structure (partly insignificant loadings of facets on factors) when cross-loadings were accounted for in ESEM models. Shorter versions of the FFNI, which did not exhibit these problems, might thus be preferred over the original FFNI-LF. In situations where hierarchical modeling or facet-level information are desired, the FFNI-SF and the newly devised BF (discussed below) seem good choices. When a concise measure of the three-factor model scores is needed, the SSF also depicts this structure well (which was not generally the case within the two-factor model).

Regarding the question whether two- or three-factor models of the FFNI should be preferred, our data show that both are generally valid, though the three-factor solution showed slightly better fit in most cases (see Table 3). These differences were, however, subtle, and the unequal number of facets assigned to the factors of both models (11 grandiose vs. 4 vulnerable / 4 agentic, 8 antagonistic, and 3 neurotic) renders empirical comparisons of the factorial structures unbalanced. Nonetheless, external validity evidence, as discussed below, shows that three-factor model scores are in many instances more informative than two-factor model scores (which conflate different aspects), therefore the three-factor solution of the FFNI might generally be preferable in contexts where different aspects might otherwise be intermingled (Back, 2018), or it might be used in conjunction with the two-factor model to parse the broader factors down into their constituent components (Miller et al., 2016).

### Convergent Validity

The validity evidence for the different FFNI factors observed here was generally in line with our hypotheses, derived from previous studies, and extend the nomological network of the FFNI in different aspects. We observed high correlations of the FFNI two-factor model scores with other measures of grandiose (NPI: .62 ≤ *r* ≤ .74) and vulnerable narcissism (HSNS, PNI vulnerable: .60 ≤ *r* ≤ .75). Estimates were similar to those obtained for the original English version (Glover et al., 2012). Within the three-factor model, FFNI agentic narcissism displayed the highest correlations with other agentic narcissism scales (NPI leadership/authority and grandiose exhibitionism, NARQ admiration: .45 ≤ *r* ≤ .69), and FFNI antagonistic narcissism was primarily associated with antagonistic narcissism scales (NPI entitlement/exploitativeness and NARQ rivalry: .45 ≤ *r* ≤ .67). Neurotic narcissism correlated most strongly with PNI vulnerability (.58 ≤ *r* ≤ .71), particularly its *contingent self-esteem* facet (.67 ≤ *r* ≤ .79). Generally, estimates were similar to previous studies (Miller et al., 2016; Rogoza et al., 2021). As expected, grandiose and vulnerable narcissism were substantially related to psychological entitlement, and this correlation was strongest for antagonistic aspects, confirming their central role in the structure of narcissism within the FFM (Weiss et al., 2019). Neurotic narcissism was not or only weakly related to entitlement in this study, and also only weakly related to the entitlement/exploitativeness factor of the NPI. It was related, however, to aspects of NARQ rivalry (*aggressiveness*). FFNI neurotic narcissism might thus not relate to core narcissistic characteristics by itself, but rather reflect personality dysfunction (see Miller, Lynam, Vize, et al., 2018).

The FFNI three-factor scores displayed the expected pattern of associations with nonclinical (NEO-PI-R) and clinical (PID-5) FFM measures: agentic narcissism correlated most strongly with NEO-PI-R extraversion (particularly the *assertiveness* facet), but not substantially with PID-5 detachment (likely due to its focus on extreme introversion). FFNI agentic narcissism also correlated with NEO-PI-R openness and conscientiousness (most notably *achievement striving*), and correlated negatively with agreeableness (most notably *modesty*). This pattern shows that the agentic narcissism factor does capture the agency / social dominance—aspect of extraversion, and is only weakly related to the aspect more characteristic of positive emotionality (for aspects, see DeYoung et al., 2007). This is somewhat more pronounced for short than longer versions of the FFNI (see below). Antagonistic narcissism, as expected, displayed strong associations with NEO-PI-R agreeableness (and all of its facets) as well as PID-5 antagonism. Beyond that, antagonistic narcissism was associated with single NEO-PI-R facets (most notably the *angry hostility* facet of neuroticism) and, interestingly, moderately associated with the PID-5 dimensions psychoticism, disinhibition, and detachment. FFNI neuroticism, as expected, correlated strongly with NEO-PI-R neuroticism, and with PID-5 negative affect. Beyond that, correlations with introversion/detachment were evident, and also with conscientiousness (*competence* and *self-discipline*) / disinhibition. As described above, FFNI neurotic narcissism was not directly related to scales assessing entitlement. It was even slightly *positively* associated with the NEO-PI-R facets *modesty* and *compliance*. Negative associations with facets of agreeableness were only evident for *trust*. FFNI neurotic narcissism was also not associated with PID-5 antagonism. Together, this points to FFNI neurotic narcissism assessing an emotionally instable but not entitled, socially anxious yet conforming personality. As Miller and colleagues (2016) put it, “individuals with high scores on the FFNI Neuroticism [. . .] would not likely be seen as prototypically narcissistic” (p. 13), but only the combination with the antagonistic factor would make the FFNI neuroticism factor narcissistic. However, neurotic narcissism was related substantially to aspects of NARQ rivalry and almost all PNI scales. From a process-based view, neurotic narcissism can be conceived an “exit strategy” when agentic and antagonistic modes fail in maintaining a grandiose self (Back, 2018).

### Nomological Network

We hypothesized that grandiose narcissism would be indicative of approach motivation (e.g., Krizan & Herlache, 2018; Spencer et al., 2018) and egotistic but largely adaptive functioning (e.g., Kaufman et al., 2020), yet also be related to interpersonal problems and aggression (particularly its antagonistic aspects; e.g., Vize et al., 2019). The data largely confirmed these expectations for the associations of grandiose dimension and its constituent factors with approach and avoidance motivation (Spencer et al., 2018), hubristic pride (Kaufman & Jauk, 2020), a cold-domineering interpersonal style (Miller, Gentile, & Campbell, 2013), love styles (though we did not observe a correlation with obsessive love, but instead with pragmatic love; Miller, Gentile, & Campbell, 2013), attachment (Miller, Gentile, & Campbell, 2013), and verbal and physical aggression. As further expected, grandiose narcissism was associated positively with self-esteem and life satisfaction (Jauk & Kaufman, 2018; Miller et al., 2016) and negatively with depression.

FFNI grandiose narcissism—primarily its antagonistic, but also its agentic aspects—further correlated with a borderline personality disorder screening measure (MSI-BPD) and with a more fine-grained measure of personality functioning (IPO). This is indicative of impairments in self- and other-related perceptual and regulatory capacities (APA, 2013) which seem to be not exclusively related to vulnerable/neurotic aspects of narcissism (Miller, Lynam, Vize, et al., 2018) but also to grandiose ones. It has long been stressed that narcissism is related to reduced personality functioning, and that the level of functioning moderates the degree of pathology associated with narcissism (Kernberg, 1975). The correlations show that this association is of sizable degree. The IPO subscales further discriminated between neurotic and antagonistic aspects of narcissism; the former being associated with *identity diffusion*, the latter with *primitive defenses* and *impaired reality testing*. Individuals high in antagonistic narcissism thus seem to be more likely to respond to self-threats with projection,^10^ for instance, and have a lower capacity to differentiate inner from outer stimuli (Lenzenweger et al., 2001). This also overlaps conceptually with the PID-5 psychoticism factor, which was also substantially correlated with FFNI antagonistic narcissism.

The impairments associated with grandiose, particularly antagonistic narcissism reflect mainly in externalizing behavior, as we found that antagonistic narcissism was associated with domineering and vindictive interpersonal problems and self-reported aggression, including physical aggression. In the offender sample, antagonistic narcissism and almost all of its facets were significantly elevated, underlining the practical relevance of antagonistic traits with respect to criminal behavior (DeLisi, 2019; Niemeyer et al., 2021, 2022).

Vulnerable narcissism, as expected, was associated with avoidance orientation (Krizan & Herlache, 2018; Spencer et al., 2018), hubristic pride (Kaufman & Jauk, 2020), interpersonal problems across the circumplex, particularly in the socially avoidant octant (Miller, Gentile, & Campbell, 2013), game-playing (ludus) and obsessive (mania) love (Miller, Gentile, & Campbell, 2013) and beyond that also pragmatic love, attachment anxiety and avoidance, and aggression (primarily anger and hostility). Also as expected, vulnerable narcissism displayed substantial negative associations with self-esteem and life satisfaction, and substantial positive associations with symptom load and depression. Comparing individuals with and without prior diagnoses of mental disorders further substantiates this in terms of elevated scores on vulnerable, specifically neurotic narcissism. Lower *indifference* and higher empathy (lower *lack of empathy*) might amplify mental health problems in these individuals in terms of over-involvement and empathic distress (cf. Singer & Klimecki, 2014). Vulnerable narcissism was further accompanied by considerable impairments in personality functioning, as evident in the MSI-BPD and the IPO. In the latter, neurotic narcissism was related to *identity diffusion*, suggesting that functioning deficits in neurotic narcissism are primarily found in a weakly integrated self-concept.

### Brief Form

By using the metaheuristic selection algorithm ACO (Olaru et al., 2015, 2019; Schroeders et al., 2016), derived a brief form that yielded satisfactory model fit for the three-factor model, good reliability, and high correlations with the FFNI-SF, on which item selection was based. The internal structure was optimized not only with respect to overall fit and factor saturation (rendering the use of ESEM unnecessary for the FFNI-BF), but also with respect to convergent validity with other narcissism measures. The FFNI-BF thus provides an economic and at the same time psychometrically optimized, comprehensive assessment of the three-factor model (however, it can be used also within the two-factor model). Correlation patterns with validity indicators were highly similar for the FFNI-LF/SF and the brief form. The FFNI-BF discriminated between groups of individuals with and without diagnoses of mental disorders and incarceration status not only at factor but also at facet level similarly to longer versions, making it a potentially useful assessment tool for clinicians or researchers who are interested in FFNI profiles in clinical or forensic groups. Taken together, the FFNI-BF is a viable inventory covering the complete profile of agentic, antagonistic, and neurotic narcissism.

Some differences to the longer versions were evident for the agentic narcissism factor, which (1) correlated less strongly with NEO-PI-R extraversion facets related to positive emotionality (rather than social dominance; similar for the SSF), (2) shifted somewhat more toward the maladaptive pole in correlations with measures of psychological adjustment, and (3) correlated somewhat more strongly with PID-5 psychoticism, as well as aspects of the PNI. While the differences are subtle, they suggest that short versions of the FFNI agentic narcissism factor—constructed using different approaches each selecting a best-performing set of items—emphasize maladaptive aspects slightly more than longer versions. One advantage of this might be that the short scales seem to capture agentic-extraverted narcissism, not just extraversion per se, to a larger degree.

A similar point applies to the vulnerable narcissism factor of the FFNI-BF, which—compared to the recently developed FFNI-SSF—shows a more substantial correlation with measures of entitlement (PES and NPI entitlement/exploitativeness) and disagreeableness/antagonism (NEO-PI-R and PID-5), similar to the longer scale versions. Since entitlement/antagonism are regarded as core characteristics of narcissism (Krizan & Herlache, 2018; Weiss et al., 2019), the stronger saturation of the FFNI-BF with entitlement/antagonism might be regarded as an advantage comparing to the FFNI-SSF. However, future research will be needed to directly compare these scale versions.

### Future Directions

While the research reported here provides promising evidence for the internal consistency, factor structure, and validity of the German FFNI and the novel FFNI-BF, there are limitations. First, validity evidence presented here relies mainly on self-report measures, which can be biased in the direction of more favorable self-presentations in grandiose narcissism (cf. Jauk et al., 2016; Mota et al., 2019; Oltmanns et al., 2018). Future research could study the relations between, self-, peer-, and expert assessments of narcissism and their validity with respect to more objective criteria. However, FFNI scales have previously been shown to relate substantially to interview-based assessments of NPD (Miller, Few, et al., 2013), and information for the clinical and forensic context presented here supports its validity with respect to more objective criteria. Second and relatedly, while the evidence on personality functioning presented here might give impulses to systematically study different aspects of narcissism from the perspective of personality functioning, it was also limited to self-report measures. These could be complemented by expert assessments of personality functioning or performance-based measures of emotional competencies, which can be seen as proxies (Jauk & Ehrenthal, 2021). Finally, the psychometric evaluation of the FFNI-BF and FFNI-SSF provided here were based on samples whose individuals filled in longer scale versions, and internal consistencies could be lower when directly administering short scales. However, since the items of the FFNI scales are presented intermixed rather than facet-wise, the effects might not be particularly strong.

### Conclusions

We validated the FFNI in German-speaking samples and constructed a novel 30-item brief form. The FFNI generally displayed good internal consistency and convergent validity with other narcissism measures. Regarding its internal structure, we found that existing short versions align with the hypothesized factor structure when an adequate level of model complexity is taken into account. Guided by criterion-based optimization, we constructed a novel brief form which clearly depicts the factorial structure with a small item set, yet is more strongly saturated with theoretically relevant core characteristics. Regarding external validity, grandiose narcissism—particularly its agentic aspects—displayed correlations with validity measures indicative of egotistical but largely adaptive adjustment, but also impaired personality functioning. Vulnerable narcissism—particularly its neurotic but also its antagonistic aspects—was associated with maladaptive adjustment and markedly reduced personality functioning. Antagonistic and neurotic narcissism effectively discriminate between groups with and without a clinical and forensic history. We conclude that the German FFNI is a reliable and valid instrument for assessing narcissism from an FFM perspective, major strengths of which lie in its breadth spanning both grandiose and vulnerable aspects encompassing agentic, antagonistic, and neurotic narcissism, and the compatibility with general nonclinical and clinical personality models. The FFNI provides a comprehensive tool for assessing the distinct aspects of narcissism in a variety of research and applied contexts.

## Supplemental Material

sj-docx-1-asm-10.1177_10731911221075761 – Supplemental material for Validation of the German Five-Factor Narcissism Inventory and Construction of a Brief Form Using Ant Colony Optimization Click here for additional data file. Supplemental material, sj-docx-1-asm-10.1177_10731911221075761 for Validation of the German Five-Factor Narcissism Inventory and Construction of a Brief Form Using Ant Colony Optimization by Emanuel Jauk, Gabriel Olaru, Eva Schürch, Mitja D. Back and Carolyn C. Morf in Assessment

## Notes

1. Note that while Wink (1991) also used the terms overt and covert narcissism to describe the two dimensions, grandiose and vulnerable appear to be more conceptually precise (Pincus & Lukowitsky, 2010).

2. Note that while Miller and colleagues (2016) used the terms agentic extraversion, antagonism, and neuroticism to describe the three factors, we speak of agentic, antagonistic, and neurotic *narcissism* here to avoid terminological confusion with general FFM factors.

3. We note that this 4-item scale displayed a low internal consistency of α = .41, which is similar to the original study (.44 < α < .47; Ackerman et al., 2011). Ackerman and colleagues provide a discussion of why the score can be informative irrespective of its low internal consistency.

4. Note that the *entitlement rage* subscale was assigned to the grandiosity and vulnerability factors in different studies. Here, we assign it to the grandiose factor (see also Morf et al., 2017).

5. ESESM-within-CFA is a two-step approach in which an ESEM model for the lower-order part of a hierarchical model is first estimated separately, and the parameter estimates of this model are then used as starting values for the respective part of a joint hierarchical CFA model. For identification purposes, *m*^2^ parameters have to be constrained, where *m* represents the number of factors in the ESEM part (Morin & Asparouhov, 2018). We estimated the ESEM models for the FFNI facets (correlated facets model, see Table 3) and then, in the joint CFA models, constrained the facet variances to 1 and the lowest cross-loadings per facet to their starting values from the ESEM solution.

6. While the optimization criteria for extraversion and antagonism are conceptually based on the three-factor model, such criteria are not currently available for neurotic narcissism; therefore, we used measures of vulnerable narcissism (two-factor model) here.

7. Content Redundancy was determined on a consensual basis by the authors. Crossed-out items were removed because of a high overlap with the other listed item: *Exhibitionism* FFNI140; *Grandiose Fantasy* FFNI037; *Indifference* FFNI123; *Lack of Empathy* FFNI146; *Manipulativeness* FFNI053; *Reactive Anger* FFNI046.

8.
FFNI060 FFNI064

9. For comparability, we evaluated two- and three-factor models (though the optimization was based on the three-factor model) and used Maximum Likelihood estimation, as in Study 1.

10. See Kampe et al. (2021) for a recent and fine-grained analysis of defense styles in narcissism.

## References

[bibr1-10731911221075761] Ackerman R. A. Donnellan M. B. Wright A. G. C. (2019). Current conceptualizations of narcissism:. Current Opinion in Psychiatry, 32(1), 32–37. 10.1097/YCO.0000000000000463 30234526

[bibr2-10731911221075761] Ackerman R. A. Witt E. A. Donnellan M. B. Trzesniewski K. H. Robins R. W. Kashy D. A. (2011). What does the narcissistic personality inventory really measure? Assessment, 18(1), 67–87. 10.1177/1073191110382845 20876550

[bibr3-10731911221075761] American Psychiatric Association. (1980). Diagnostic and statistical manual of mental disorders (3rd ed.).

[bibr4-10731911221075761] American Psychiatric Association. (1994). Diagnostic and statistical manual of mental disorders (4th ed.).

[bibr5-10731911221075761] American Psychiatric Association. (2013). Diagnostic and statistical manual of mental disorders (5th ed.). American Psychiatric Publishing. 10.1176/appi.books.9780890425596

[bibr6-10731911221075761] Angleitner A. Ostendorf F. Riemann R. (2011). DAPP-BQ: Dimensional Assessment of Personality Pathology–Basic Questionnaire. Department of Psychology, Universität Bielefeld.

[bibr7-10731911221075761] Asparouhov T. Muthén B. (2009). Exploratory structural equation modeling. Structural Equation Modeling: A Multidisciplinary Journal, 16(3), 397–438. 10.1080/10705510903008204

[bibr8-10731911221075761] Back M. D. (2018). The narcissistic admiration and rivalry concept. In Hermann A. D. Brunell A. B. Foster J. D. (Eds.), Handbook of trait narcissism (pp. 57–67). Springer. 10.1007/978-3-319-92171-6_6

[bibr9-10731911221075761] Back M. D. Küfner A. C. P. Dufner M. Gerlach T. M. Rauthmann J. F. Denissen J. J. A. (2013). Narcissistic admiration and rivalry: Disentangling the bright and dark sides of narcissism. Journal of Personality and Social Psychology, 105(6), 1013–1037. 10.1037/a0034431 24128186

[bibr10-10731911221075761] Bagby R. M. Farvolden P. (2004). The Personality Diagnostic Questionnaire-4 (PDQ-4). In Hilsenroth M. J. Segal D. L. (Eds.), Comprehensive handbook of psychological assessment, Vol. 2. Personality assessment (pp. 122–133). John Wiley.

[bibr11-10731911221075761] Bender D. S. Morey L. C. Skodol A. E. (2011). Toward a model for assessing level of personality functioning in DSM–5, part I: A review of theory and methods. Journal of Personality Assessment, 93(4), 332–346. 10.1080/00223891.2011.583808 22804672

[bibr12-10731911221075761] Bierhoff H. W. Grau I. Ludwig A. (1993). Marburger Einstellungs-Inventar für Liebesstile [Marburg Love Attitudes Scale]. Hogrefe.

[bibr13-10731911221075761] Brenk-Franz K. Ehrenthal J. Freund T. Schneider N. Strauß B. Tiesler F. Schauenburg H. Gensichen J. (2018). Evaluation of the short form of “Experience in Close Relationships” (Revised, German Version “ECR-RD12”)—A tool to measure adult attachment in primary care. PLOS ONE, 13(1), Article e0191254. 10.1371/journal.pone.0191254 PMC5779670 29360832

[bibr14-10731911221075761] Brioschi S. Franchini L. Fregna L. Borroni S. Franzoni C. Fossati A. Colombo C. (2020). Clinical and personality profile of depressed suicide attempters: A preliminary study at the open-door policy Mood Disorder Unit of San Raffaele Hospital. Psychiatry Research, 287, 112575. 10.1016/j.psychres.2019.112575 31587915

[bibr15-10731911221075761] Buss D. M. Chiodo L. M. (1991). Narcissistic acts in everyday life. Journal of Personality, 59(2), 179–215. 10.1111/j.1467-6494.1991.tb00773.x 1880699

[bibr16-10731911221075761] Campbell W. K. Bonacci A. M. Shelton J. Exline J. J. Bushman B. J. (2004). Psychological entitlement: Interpersonal consequences and validation of a self-report measure. Journal of Personality Assessment, 83(1), 29–45. 10.1207/s15327752jpa8301_04 15271594

[bibr17-10731911221075761] Carver C. S. White T. L. (1994). Behavioral inhibition, behavioral activation, and affective responses to impending reward and punishment: The BIS/BAS Scales. Journal of Personality and Social Psychology, 67(2), 319–333. 10.1037/0022-3514.67.2.319

[bibr18-10731911221075761] Chester D. S. Lasko E. N. (2019). Validating a standardized approach to the Taylor aggression paradigm. Social Psychological and Personality Science, 10(5), 620–631. 10.1177/1948550618775408

[bibr19-10731911221075761] Collison K. L. Vize C. E. Miller J. D. Lynam D. R. (2018). Development and preliminary validation of a five factor model measure of Machiavellianism. Psychological Assessment, 30(10), 1401–1407. 10.1037/pas0000637 30047746

[bibr20-10731911221075761] Costa P. T. McCrae R. R. (1992). Neo Personality Inventory-Revised (NEO PI-R). Psychological Assessment Resources.

[bibr21-10731911221075761] Crowe M. L. Lynam D. R. Campbell W. K. Miller J. D. (2019). Exploring the structure of narcissism: Toward an integrated solution. Journal of Personality, 87(6), 1151–1169. 10.1111/jopy.12464 30742713

[bibr22-10731911221075761] Dai Q. Shimotsukasa T. Oshio A. (2021). Short form of the Five-Factor Narcissism Inventory: A Japanese adaptation. Cogent Psychology, 8(1), 1935533. 10.1080/23311908.2021.1935533

[bibr23-10731911221075761] DeLisi M. (2019). Antagonism and crime. In *The* handbook of antagonism (pp. 297–309). Elsevier. 10.1016/B978-0-12-814627-9.00020-7

[bibr24-10731911221075761] Derogatis L. R. Lazarus L. (1994). SCL-90-R, Brief Symptom Inventory, and matching clinical rating scales. In Maruish M. E. (Ed.), The use of psychological testing for treatment planning and outcome assessment (pp. 217–248). Lawrence Erlbaum.

[bibr25-10731911221075761] Derogatis L. R. Melisaratos N. (1983). The Brief Symptom Inventory: An introductory report. Psychological Medicine, 13(3), 595–605. 10.1017/S0033291700048017 6622612

[bibr26-10731911221075761] DeYoung C. G. Quilty L. C. Peterson J. B. (2007). Between facets and domains: 10 aspects of the Big Five. Journal of Personality and Social Psychology, 93(5), 880–896. 10.1037/0022-3514.93.5.880 17983306

[bibr27-10731911221075761] Diamond P. M. Magaletta P. R. (2006). The Short-Form Buss-Perry Aggression Questionnaire (BPAQ-SF): A validation study with federal offenders. Assessment, 13(3), 227–240. 10.1177/1073191106287666 16880276

[bibr28-10731911221075761] Diener E. Emmons R. A. Larsen R. J. Griffin S. (1985). The Satisfaction With Life Scale. Journal of Personality Assessment, 49(1), 71–75. 10.1207/s15327752jpa4901_13 16367493

[bibr29-10731911221075761] Di Sarno M. Zimmermann J. Madeddu F. Casini E. Di Pierro R . (2020). Shame behind the corner? A daily diary investigation of pathological narcissism. Journal of Research in Personality, 85, 103924. 10.1016/j.jrp.2020.103924

[bibr30-10731911221075761] Fossati A. Borroni S. Grazioli F. Dornetti L. Marcassoli I. Maffei C. Cheek J. (2009). Tracking the hypersensitive dimension in narcissism: Reliability and validity of the Hypersensitive Narcissism Scale. Personality and Mental Health, 3(4), 235–247. 10.1002/pmh.92

[bibr31-10731911221075761] Fossati A. Somma A. Borroni S. Miller J. D. (2018). Assessing dimensions of pathological narcissism: Psychometric properties of the short form of the Five-Factor Narcissism Inventory in a sample of Italian University students. Journal of Personality Assessment, 100(3), 250–258. 10.1080/00223891.2017.1324457 28537767

[bibr32-10731911221075761] Fraley R. C. Waller N. G. Brennan K. A. (2000). An item response theory analysis of self-report measures of adult attachment. Journal of Personality and Social Psychology, 78(2), 350–365. 10.1037/0022-3514.78.2.350 10707340

[bibr33-10731911221075761] Franke G. H. (2000). Brief Symptom Inventory von L. R. Derogatis (Kurzform der SCL-90- R). Beltz Test.

[bibr34-10731911221075761] Glaesmer H. Grande G. Braehler E. Roth M. (2011). The German Version of the Satisfaction With Life Scale (SWLS): Psychometric properties, validity, and population-based norms. European Journal of Psychological Assessment, 27(2), 127–132. 10.1027/1015-5759/a000058

[bibr35-10731911221075761] Glover N. Miller J. D. Lynam D. R. Crego C. Widiger T. A. (2012). The Five-Factor Narcissism Inventory: A five-factor measure of narcissistic personality traits. Journal of Personality Assessment, 94(5), 500–512. 10.1080/00223891.2012.670680 22475323

[bibr36-10731911221075761] Grapsas S. Brummelman E. Back M. D. Denissen J. J. A. (2020). The “why” and “how” of narcissism: A process model of narcissistic status pursuit. Perspectives on Psychological Science, 15(1), 150–172. 10.1177/1745691619873350 31805811 PMC6970445

[bibr37-10731911221075761] Gray J. A. (1970). The psychophysiological basis of introversion-extraversion. Behaviour Research and Therapy, 8(3), 249–266. 10.1016/0005-7967(70)90069-0 5470377

[bibr38-10731911221075761] Hart W. Tortoriello G. K. Breeden C. J. (2020). Entitled due to deprivation vs. superiority: Evidence that unidimensional entitlement scales blend distinct entitlement rationales across psychological dimensions. Journal of Personality Assessment, 102(6), 781–791. 10.1080/00223891.2019.1674319 31618099

[bibr39-10731911221075761] Hautzinger M. Bailer M. (1993). Allgemeine Depressions Skala. Manual [Manual of the German version of the Center for Epidemiologic Studies Depression Scale]. Beltz Test.

[bibr40-10731911221075761] Helle A. C. Mullins-Sweatt S. N. (2019). Maladaptive personality trait models: Validating the five-factor model maladaptive trait measures with the personality inventory for DSM-5 and NEO personality inventory. Assessment, 26(3), 375–385. 10.1177/1073191117709071 28503932

[bibr41-10731911221075761] Hendin H. M. Cheek J. M. (1997). Assessing hypersensitive narcissism: A reexamination of Murray’s Narcism Scale. Journal of Research in Personality, 31(4), 588–599. 10.1006/jrpe.1997.2204

[bibr42-10731911221075761] Hendrick C. Hendrick S. (1986). A theory and method of love. Journal of Personality and Social Psychology, 50(2), 392–402. 10.1037/0022-3514.50.2.392

[bibr43-10731911221075761] Horowitz L. M. Rosenberg S. E. Baer B. A. Ureño G. Villaseñor V. S. (1988). Inventory of interpersonal problems: Psychometric properties and clinical applications. Journal of Consulting and Clinical Psychology, 56(6), 885–892. 10.1037//0022-006X.56.6.885 3204198

[bibr44-10731911221075761] Hyatt C. S. Chester D. S. Zeichner A. Miller J. D. (2019). Analytic flexibility in laboratory aggression paradigms: Relations with personality traits vary (slightly) by operationalization of aggression. Aggressive Behavior, 45(4), 377–388. 10.1002/ab.21830 30852848

[bibr45-10731911221075761] Infante A. A. Wang X. Pardini D. (2019). The development and validation of a multidimensional scale of perceived Latino threat. Journal of Ethnic and Migration Studies. Advance online publication. 10.1080/1369183X.2019.1616539

[bibr46-10731911221075761] Jauk E. Ehrenthal J. C. (2021). Self-reported levels of personality functioning from the Operationalized Psychodynamic Diagnosis (OPD) system and emotional intelligence likely assess the same latent construct. Journal of Personality Assessment, 103, 365–379. 10.1080/00223891.2020.1775089 32631173 PMC7611281

[bibr47-10731911221075761] Jauk E. Freudenthaler H. H. Neubauer A. C. (2016). The dark triad and trait versus ability emotional intelligence: Emotional darkness differs between women and men. Journal of Individual Differences, 37(2), 112–118. 10.1027/1614-0001/a000195

[bibr48-10731911221075761] Jauk E. Kaufman S. B. (2018). The higher the score, the darker the core: The nonlinear association between grandiose and vulnerable narcissism. Frontiers in Psychology, 9, Article 1305. 10.3389/fpsyg.2018.01305 PMC6088174 30150950

[bibr49-10731911221075761] Jauk E. Ulbrich L. Jorschick P. Höfler M. Kaufman S. B. Kanske P. (2021). The nonlinear association between grandiose and vulnerable narcissism: An individual data meta-analysis. Journal of Personality. Advance online publication. 10.1111/jopy.12692 34860434

[bibr50-10731911221075761] Jauk E. Weigle E. Lehmann K. Benedek M. Neubauer A. C. (2017). The relationship between grandiose and vulnerable (hypersensitive) narcissism. Frontiers in Psychology, 8, Article 1600. 10.3389/fpsyg.2017.01600 PMC5601176 28955288

[bibr51-10731911221075761] Jones D. N. Paulhus D. L. (2014). Introducing the Short Dark Triad (SD3): A brief measure of dark personality traits. Assessment, 21(1), 28–41. 10.1177/1073191113514105 24322012

[bibr52-10731911221075761] Kampe L. Bohn J. Remmers C. Hörz-Sagstetter S. (2021). It’s not that great anymore: The central role of defense mechanisms in grandiose and vulnerable narcissism. Frontiers in Psychiatry, 12, Article 661948. 10.3389/fpsyt.2021.661948 PMC8226035 34177651

[bibr53-10731911221075761] Kaufman S. B. Jauk E. (2020). Healthy selfishness and pathological altruism: Measuring two paradoxical forms of selfishness. Frontiers in Psychology, 11, Article 1006. 10.3389/fpsyg.2020.01006 PMC7265883 32528378

[bibr54-10731911221075761] Kaufman S. B. Weiss B. Miller J. D. Campbell W. K. (2020). Clinical correlates of vulnerable and grandiose narcissism: A personality perspective. Journal of Personality Disorders, 34, 107–130. 10.1521/pedi_2018_32_384 30179576

[bibr55-10731911221075761] Kernberg O. F. (1975). Borderline conditions and pathological narcissism. Aronson.

[bibr56-10731911221075761] Krizan Z. Herlache A. D. (2018). The narcissism spectrum model: A synthetic view of narcissistic personality. Personality and Social Psychology Review, 22(1), 3–31. 10.1177/1088868316685018 28132598

[bibr57-10731911221075761] Kröger C. Vonau M. Kliem S. Kosfelder J. (2010). Screening-Instrument für die Borderline-Persönlichkeitsstörung. Diagnostische Effizienz der deutschen Version des McLean Screening Instrument for Borderline Personality Disorder. PPmP—Psychotherapie · Psychosomatik · Medizinische Psychologie, 60(09/10), 391–396. 10.1055/s-0030-1248279 20225166

[bibr58-10731911221075761] Krueger R. F. Derringer J. Markon K. E. Watson D. Skodol A. E. (2012). Initial construction of a maladaptive personality trait model and inventory for DSM-5. Psychological Medicine, 42(9), 1879–1890. 10.1017/S0033291711002674 22153017 PMC3413381

[bibr59-10731911221075761] Lenzenweger M. F. Clarkin J. F. Kernberg O. F. Foelsch P. A. (2001). The Inventory of Personality Organization: Psychometric properties, factorial composition, and criterion relations with affect, aggressive dyscontrol, psychosis proneness, and self-domains in a nonclinical sample. Psychological Assessment, 13(4), 577–591. 11793901

[bibr60-10731911221075761] Livesley W. J. Jackson D. (2009). Manual for the dimensional assessment of personality pathology: Basic questionnaire. Sigma Assessment Systems.

[bibr61-10731911221075761] Lynam D. R. Gaughan E. T. Miller J. D. Miller D. J. Mullins-Sweatt S. Widiger T. A. (2011). Assessing the basic traits associated with psychopathy: Development and validation of the elemental psychopathy assessment. Psychological Assessment, 23(1), 108–124. 10.1037/a0021146 21171784

[bibr62-10731911221075761] Mazinani Z. Shakiba S. Pourshahbaz A. Vahedi M. (2021). Five Factor Narcissism and threat to fundamental needs following social exclusion engendered by the Cyberball game. Personality and Individual Differences, 168, 110279. 10.1016/j.paid.2020.110279

[bibr63-10731911221075761] Miller J. D. Few L. R. Wilson L. Gentile B. Widiger T. A. Mackillop J. Keith Campbell W. (2013). The Five-Factor Narcissism Inventory (FFNI): A test of the convergent, discriminant, and incremental validity of FFNI scores in clinical and community samples. Psychological Assessment, 25(3), 748–758. 10.1037/a0032536 23647044

[bibr64-10731911221075761] Miller J. D. Gentile B. Campbell W. K. (2013). A test of the construct validity of the Five-Factor Narcissism Inventory. Journal of Personality Assessment, 95(4), 377–387. 10.1080/00223891.2012.742903 23186210

[bibr65-10731911221075761] Miller J. D. Hoffman B. J. Gaughan E. T. Gentile B. Maples J. Keith Campbell W. (2011). Grandiose and vulnerable narcissism: A nomological network analysis: Variants of narcissism. Journal of Personality, 79(5), 1013–1042. 10.1111/j.1467-6494.2010.00711.x 21204843

[bibr66-10731911221075761] Miller J. D. Lynam D. R. Hyatt C. S. Campbell W. K. (2017). Controversies in narcissism. Annual Review of Clinical Psychology, 13(1), 291–315. 10.1146/annurev-clinpsy-032816-045244 28301765

[bibr67-10731911221075761] Miller J. D. Lynam D. R. McCain J. L. Few L. R. Crego C. Widiger T. A. Campbell W. K. (2016). Thinking structurally about narcissism: An examination of the Five-Factor Narcissism Inventory and its components. Journal of Personality Disorders, 30(1), 1–18. 10.1521/pedi_2015_29_177 25710734

[bibr68-10731911221075761] Miller J. D. Lynam D. R. Siedor L. Crowe M. Campbell W. K. (2018). Consensual lay profiles of narcissism and their connection to the Five-Factor Narcissism Inventory. Psychological Assessment, 30(1), 10–18. 10.1037/pas0000460 29323510

[bibr69-10731911221075761] Miller J. D. Lynam D. R. Vize C. Crowe M. Sleep C. Maples-Keller J. L. Few L. R. Campbell W. K. (2018). Vulnerable narcissism is (mostly) a disorder of neuroticism. Journal of Personality, 86(2), 186–199. 10.1111/jopy.12303 28170100

[bibr70-10731911221075761] Miller J. D. McCain J. Lynam D. R. Few L. R. Gentile B. MacKillop J. Campbell W. K. (2014). A comparison of the criterion validity of popular measures of narcissism and narcissistic personality disorder via the use of expert ratings. Psychological Assessment, 26(3), 958–969. 10.1037/a0036613 24773036

[bibr71-10731911221075761] Morey L. C. Waugh M. H. Blashfield R. K. (1985). MMPI scales for DSM-III personality disorders: Their derivation and correlates. Journal of Personality Assessment, 49(3), 245–251. 10.1207/s15327752jpa4903_5 4032202

[bibr72-10731911221075761] Morf C. C. Horvath S. Torchetti L. (2011). Narcissistic self-enhancement: Tales of (successful?) self-portrayal. In Alicke M. D. Sedikides C. (Eds.), Handbook of self-enhancement and self-protection (pp. 399–424). Guilford Press.

[bibr73-10731911221075761] Morf C. C. Rhodewalt F. (2001). Unraveling the paradoxes of narcissism: A dynamic self-regulatory processing model. Psychological Inquiry, 12(4), 177–196. 10.1207/S15327965PLI1204_1

[bibr74-10731911221075761] Morf C. C. Schürch E. Küfner A. Siegrist P. Vater A. Back M. Mestel R. Schröder-Abé M. (2017). Expanding the nomological net of the Pathological Narcissism Inventory: German validation and extension in a clinical inpatient sample. Assessment, 24(4), 419–443. 10.1177/1073191115627010 26874362

[bibr75-10731911221075761] Morin A. J. S. Asparouhov T. (2018). Estimation of a hierarchical Exploratory Structural Equation Model (ESEM) using ESEM-within-CFA. Substantive Methodological Synergy Research Laboratory.

[bibr76-10731911221075761] Mota S. Leckelt M. Geukes K. Nestler S. Humberg S. Schröder-Abé M. Schmukle S. C. Back M. D. (2019). A comprehensive examination of narcissists’ self-perceived and actual socioemotional cognition ability. Collabra: Psychology, 5(1), 6. 10.1525/collabra.174

[bibr77-10731911221075761] Niemeyer L. M. Grosz M. P. Jallalvand L. Mota S. Back M. D. (2021). Toward a differentiated assessment of narcissism in forensic contexts: Validating the Narcissistic Admiration and Rivalry Questionnaire–Short Scale (NARQ-S) in a Forensic Sample. Assessment. Advance online publication. 10.1177/1073191120986608 33538175

[bibr78-10731911221075761] Niemeyer L. M. Grosz M. P. Zimmermann J. Back M. D. (2022). Assessing maladaptive personality in the forensic context: Development and validation of the Personality Inventory for DSM-5 Forensic Faceted Brief Form (PID-5-FFBF). Journal of Personality Assessment, 104, 30–43. 10.1080/00223891.2021.1923522 34037499

[bibr79-10731911221075761] Olaru G. Schroeders U. Hartung J. Wilhelm O. (2019). Ant colony optimization and local weighted structural equation modeling. A tutorial on novel item and person sampling procedures for personality research. European Journal of Personality, 33(3), 400–419. 10.1002/per.2195

[bibr80-10731911221075761] Olaru G. Witthöft M. Wilhelm O. (2015). Methods matter: Testing competing models for designing short-scale Big-Five assessments. Journal of Research in Personality, 59, 56–68. 10.1016/j.jrp.2015.09.001

[bibr81-10731911221075761] Oltmanns J. R. Crego C. Widiger T. A. (2018). Informant assessment: The informant Five-Factor Narcissism Inventory. Psychological Assessment, 30(1), 31–42. 10.1037/pas0000487 29323512 PMC5930358

[bibr82-10731911221075761] Ostendorf F. Angleitner A . (2004). NEO-Persönlichkeitsinventar nach Costa und McCrae. Revidierte Fassung [NEO - Personality Inventory according to Costa and McCrae. Revised version]. Hogrefe.

[bibr83-10731911221075761] Packer West M. Miller J. D. Weiss B. Spencer C. C. Crowe M. L. Campbell W. K. Lynam D. R . (2021). Development and validation of the super-short form of the Five-Factor Narcissism Inventory (FFNI-SSF). Personality and Individual Differences, 177, 110825. 10.1016/j.paid.2021.110825

[bibr84-10731911221075761] Papageorgiou K. A. Denovan A. Dagnall N. (2019). The positive effect of narcissism on depressive symptoms through mental toughness: Narcissism may be a dark trait but it does help with seeing the world less grey. European Psychiatry, 55, 74–79. 10.1016/j.eurpsy.2018.10.002 30390475

[bibr85-10731911221075761] Papageorgiou K. A. Denovan A. Dagnall N. Artamonova E. (2021). A cross-cultural investigation of the Five-Factor Narcissism Inventory short form: Narcissism as a multidimensional trait in the United Kingdom and Russia. Journal of Personality Assessment. Advance online publication. 10.1080/00223891.2021.1929263 34096816

[bibr86-10731911221075761] Papageorgiou K. A. Gianniou F.-M. Wilson P. Moneta G. B. Bilello D. Clough P. J. (2019). The bright side of dark: Exploring the positive effect of narcissism on perceived stress through mental toughness. Personality and Individual Differences, 139, 116–124. 10.1016/j.paid.2018.11.004

[bibr87-10731911221075761] Paulhus D. L. (2001). Normal narcissism: Two minimalist accounts. Psychological Inquiry, 12(4), 228–230.

[bibr88-10731911221075761] Pincus A. L. Ansell E. B. Pimentel C. A. Cain N. M. Wright A. G. C. Levy K. N. (2009). Initial construction and validation of the Pathological Narcissism Inventory. Psychological Assessment, 21(3), 365–379. 10.1037/a0016530 19719348

[bibr89-10731911221075761] Pincus A. L. Dowgwillo E. A. Greenberg L. S. (2016). Three cases of narcissistic personality disorder through the lens of the DSM-5 alternative model for personality disorders. Practice Innovations, 1(3), 164–177. 10.1037/pri0000025

[bibr90-10731911221075761] Pincus A. L. Lukowitsky M. R. (2010). Pathological narcissism and narcissistic personality disorder. Annual Review of Clinical Psychology, 6(1), 421–446. 10.1146/annurev.clinpsy.121208.131215 20001728

[bibr91-10731911221075761] Prendergast C. N. Haahjem Eftedal N. Fredriksen Ikonomeas A. G. Brun A. Huth H. Bredesen M. (2019). The Norwegian version of the Five Factor Narcissism Inventory for vulnerable narcissism and the grandiose narcissism subscale of indifference: Psychometric properties of the long- and short-form versions. Scandinavian Journal of Psychology, 60(5), 492–500. 10.1111/sjop.12569 31402468

[bibr92-10731911221075761] Radloff L. S. (1977). The CES-D scale: A self-report depression scale for research in the general population. Applied Psychological Measurement, 1(3), 385–401. 10.1177/014662167700100306

[bibr93-10731911221075761] Raskin R. Hall C. S. (1979). A Narcissistic Personality Inventory. Psychological Reports, 45(2), 590–590. 10.2466/pr0.1979.45.2.590 538183

[bibr94-10731911221075761] Raskin R. Hall C. S. (1981). The Narcissistic Personality Inventory: Alternative form reliability and further evidence of construct validity. Journal of Personality Assessment, 45(2), 159–162. 10.1207/s15327752jpa4502_10 16370732

[bibr95-10731911221075761] Raskin R. Terry H. (1988). A principal-components analysis of the Narcissistic Personality Inventory and further evidence of its construct validity. Journal of Personality and Social Psychology, 54(5), 890–902. 10.1037/0022-3514.54.5.890 3379585

[bibr96-10731911221075761] Rogoza R. Cieciuch J. Strus W. Kłosowski M. (2021). Investigating the structure of the Polish Five Factor Narcissism Inventory: Support for the three-factor model of narcissism. Psychological Assessment, 33(3), 267–272. 10.1037/pas0000901 33090830

[bibr97-10731911221075761] Rosenberg M. (1965). Society and the adolescent self-image. Princeton University Press.

[bibr98-10731911221075761] Şar V. Türk-Kurtça T. (2021). The vicious cycle of traumatic narcissism and dissociative depression among young adults: A trans-diagnostic approach. Journal of Trauma & Dissociation, 22, 502–521. 10.1080/15299732.2020.1869644 33427111

[bibr99-10731911221075761] Sauls D. Zeigler-Hill V. (2020). Basic emotional systems and narcissistic personality features: What is the emotional core of narcissism? Personality and Individual Differences, 162, 110032. 10.1016/j.paid.2020.110032

[bibr100-10731911221075761] Schroeders U. Wilhelm O. Olaru G. (2016). Meta-heuristics in short scale construction: Ant colony optimization and genetic algorithm. PLOS ONE, 11(11), Article e0167110. 10.1371/journal.pone.0167110 PMC5125670 27893845

[bibr101-10731911221075761] Schütz A. Marcus B. Sellin I. (2004). Die Messung von Narzissmus als Persönlichkeitskonstrukt:. Diagnostica, 50(4), 202–218. 10.1026/0012-1924.50.4.202

[bibr102-10731911221075761] Sherman E. D. Miller J. D. Few L. R. Campbell W. K. Widiger T. A. Crego C. Lynam D. R. (2015). Development of a short form of the Five-Factor Narcissism Inventory: The FFNI-SF. Psychological Assessment, 27(3), 1110–1116. 10.1037/pas0000100 25774640

[bibr103-10731911221075761] Singer T. Klimecki O. M. (2014). Empathy and compassion. Current Biology, 24(18), R875–R878. 10.1016/j.cub.2014.06.054 25247366

[bibr104-10731911221075761] Somma A. Paulhus D. L. Borroni S. Fossati A. (2020). Evaluating the psychometric properties of the Short Dark Triad (SD3) in Italian adults and adolescents. European Journal of Psychological Assessment, 36(1), 185–195. 10.1027/1015-5759/a000499

[bibr105-10731911221075761] Spencer C. C. Foster J. D. Bedwell J. S. (2018). Structural relationships among the revised reinforcement sensitivity theory and grandiose and vulnerable narcissism. Journal of Personality Disorders, 32(5), 654–667. 10.1521/pedi_2017_31_318 28926305

[bibr106-10731911221075761] Strobel A. Beauducel A. Debener S. Brocke B. (2001). Eine deutschsprachige Version des BIS/BAS-Fragebogens von Carver und White. Zeitschrift für Differentielle und Diagnostische Psychologie, 22(3), 216–227. 10.1024//0170-1789.22.3.216

[bibr107-10731911221075761] Sullivan G. B. (2010). “We won!” Collective pride and identity-related emotion changes following a national team victory [Unpublished manuscript]. https://ubc-emotionlab.ca/wp-content/uploads/2011/12/Authentic-and-Hubristic-Pride-German-version.pdf

[bibr108-10731911221075761] Szymczak P. Sawicki A. Jaworski M. (2020). How narcissists see the social world? Trust, cynicism, and trifurcated model of narcissism. Current Psychology. Advance online publication. 10.1007/s12144-020-01215-z

[bibr109-10731911221075761] Thomas A. Brähler E. Strauß B. (2011). IIP-32: Entwicklung, Validierung und Normierung einer Kurzform des Inventars zur Erfassung interpersonaler Probleme. Diagnostica, 57(2), 68–83. 10.1026/0012-1924/a000034

[bibr110-10731911221075761] Tracy J. L. Cheng J. T. Robins R. W. Trzesniewski K. H. (2009). Authentic and hubristic pride: The affective core of self-esteem and narcissism. Self and Identity, 8(2–3), 196–213. 10.1080/15298860802505053

[bibr111-10731911221075761] Tracy J. L. Robins R. W. (2007). The psychological structure of pride: A tale of two facets. Journal of Personality and Social Psychology, 92(3), 506–525. 10.1037/0022-3514.92.3.506 17352606

[bibr112-10731911221075761] Tyrer P. Mulder R. Kim Y.-R. Crawford M. J. (2019). The development of the ICD-11 classification of personality disorders: An amalgam of science, pragmatism, and politics. Annual Review of Clinical Psychology, 15(1), 481–502. 10.1146/annurev-clinpsy-050718-095736 30601688

[bibr113-10731911221075761] Vize C. E. Collison K. L. Crowe M. L. Campbell W. K. Miller J. D. Lynam D. R. (2019). Using dominance analysis to decompose narcissism and its relation to aggression and externalizing outcomes. Assessment, 26(2), 260–270. 10.1177/1073191116685811 28064516

[bibr114-10731911221075761] Vize C. E. Collison K. L. Lynam D. R. (2020). The importance of antagonism: Explaining similarities and differences in psychopathy and narcissism’s relations with aggression and externalizing outcomes. Journal of Personality Disorders, 34(6), 842–854. 10.1521/pedi_2020_34_342 33146576

[bibr115-10731911221075761] von Collani G. Herzberg P. Y . (2003). Eine revidierte Fassung der deutschsprachigen Skala zum Selbstwertgefühl von Rosenberg. Zeitschrift für Differentielle und Diagnostische Psychologie, 24(1), 3–7. 10.1024//0170-1789.24.1.3

[bibr116-10731911221075761] von Collani G. Werner R . (2005). Self-related and motivational constructs as determinants of aggression. Personality and Individual Differences, 38(7), 1631–1643. 10.1016/j.paid.2004.09.027

[bibr117-10731911221075761] Wehner C. Maaß U. Leckelt M. Back M. D. Ziegler M. (2021). Validation of the Short Dark Triad in a German sample: Structure, nomological network, and an ultrashort version. European Journal of Psychological Assessment, 37, 397–408. 10.1027/1015-5759/a000617

[bibr118-10731911221075761] Weiss B. Campbell W. K. Lynam D. R. Miller J. D. (2019). A trifurcated model of narcissism: On the pivotal role of trait antagonism. In The handbook of antagonism (pp. 221–235). Elsevier. 10.1016/B978-0-12-814627-9.00015-3

[bibr119-10731911221075761] Welker L. E. Simons R. M. Simons J. S. (2019). Grandiose and vulnerable narcissism: Associations with alcohol use, alcohol problems and problem recognition. Journal of American College Health, 67(3), 226–234. 10.1080/07448481.2018.1470092 29952731 PMC6310677

[bibr120-10731911221075761] Wink P. (1991). Two faces of narcissism. Journal of Personality and Social Psychology, 61(4), 590–597. 10.1037/0022-3514.61.4.590 1960651

[bibr121-10731911221075761] Wright A. G. Edershile E. A. (2018). Issues resolved and unresolved in pathological narcissism. Current Opinion in Psychology, 21, 74–79. 10.1016/j.copsyc.2017.10.001 29059578

[bibr122-10731911221075761] Zanarini M. C. Vujanovic A. A. Parachini E. A. Boulanger J. L. Frankenburg F. R. Hennen J. (2003). A screening measure for BPD: The McLean Screening Instrument for Borderline Personality Disorder (MSI-BPD). Journal of Personality Disorders, 17(6), 568–573. 10.1521/pedi.17.6.568.25355 14744082

[bibr123-10731911221075761] Zeigler-Hill V. Sauls D. Ochoa V. Kopitz J. Besser A. (2021). Narcissism and motives to pursue status through the use of dominance-based strategies, prestige-based strategies, and leadership-based strategies. Evolutionary Psychological Science, 7, 254–272. 10.1007/s40806-021-00278-w

[bibr124-10731911221075761] Zimmermann J. Altenstein D. Krieger T. Holtforth M. G. Pretsch J. Alexopoulos J. Spitzer C. Benecke C. Krueger R. F. Markon K. E. Leising D. (2014). The structure and correlates of self-reported DSM-5 maladaptive personality traits: Findings from two German-speaking samples. Journal of Personality Disorders, 28(4), 518–540. 10.1521/pedi_2014_28_130 24511899

[bibr125-10731911221075761] Zimmermann J. Benecke C. Hörz S. Rentrop M. Peham D. Bock A. Wallner T. Schauenburg H. Frommer J. Huber D. Clarkin J. F. Dammann G. (2013). Validierung einer deutschsprachigen 16-Item-Version des Inventars der Persönlichkeitsorganisation (IPO-16). Diagnostica, 59(1), 3–16. 10.1026/0012-1924/a000076

[bibr126-10731911221075761] Zimmermann J. Brakemeier E.-L. Benecke C. (2015). Alternatives DSM-5-Modell zur Klassifikation von Persönlichkeitsstörungen: Bezüge zu psychodynamischer und verhaltenstherapeutischer Diagnostik. Psychotherapeut, 60(4), 269–279. 10.1007/s00278-015-0033-8

