# Supplementary material for: Validation of the German Five-Factor Narcissism Inventory and Construction of a Brief Form Using Ant Colony Optimization
Source: Assessment. 2022 Feb 18;30(4):969–97. doi: 10.1177/10731911221075761 (PMC10149890; doi:10.1177/10731911221075761)
Supplement: sj-docx-1-asm-10.1177_10731911221075761 – Supplemental material for Validation of the German Five-Factor Narcissism Inventory and Construction of a Brief Form Using Ant Colony Optimization [file sj-docx-1-asm-10.1177_10731911221075761.docx]

Table S1. Items and loadings of the FFNI-Brief Form (FFNI-BF).

| BF Item No. | LF Item No. | German Text | English Text | Loading |
| --- | --- | --- | --- | --- |
| **Agentic Narcissism** | | | | |
| *Acclaim Seeking* | | | | *.86* |
| 1 | 73 | Ich strebe nach Größe. | I aspire for greatness. | .85 |
| 16 | 133 | Ich bin angetrieben davon, erfolgreich zu sein. | I am driven to succeed. | .70 |
| *Authoritativeness* | | | | *.56* |
| 3 | 66 | Ich fühle mich wohl dabei, Autoritätspositionen einzunehmen. | I am comfortable taking on positions of authority. | .82 |
| 18 | 111 | Ich neige dazu, in den meisten Situationen das Kommando zu übernehmen. | I tend to take charge of most situations. | .73 |
| *Grandiose Fantasies* | | | | *.86* |
| 8 | 37 | Ich male mir oft aus, eines Tages berühmt zu sein. | I often fantasize about someday being famous. | .62 |
| 23 | 52 | Ich male mir oft aus, viel Erfolg und Macht zu haben. | I often fantasize about having lots of success and power. | .92 |
| *Exhibitionism* | | | | *.60* |
| 6 | 35 | Ich mag es, von anderen bemerkt zu werden. | I like being noticed by others. | .58 |
| 21 | 50 | Ich mag es, die beliebteste Person auf einer Party zu sein. | I like being the most popular person at a party. | .76 |
| **Antagonistic Narcissism** | | | | |
| *Manipulativeness* | | | | *.68* |
| 11 | 53 | Ich bin ziemlich gut darin, andere zu manipulieren. | I’m pretty good at manipulating people. | .75 |
| 26 | 113 | Ich kann mich in alles hinein und aus allem heraus reden. | I can talk my way into and out of anything. | .63 |
| *Exploitativeness* | | | | *.80* |
| 7 | 84 | Manchmal muss man andere benutzen, um erfolgreich zu sein. | Sometimes to succeed you need to use other people. | .78 |
| 22 | 114 | Ich bin dazu bereit, andere auszunutzen, um meine eigenen Ziele voranzubringen. | I’m willing to exploit others to further my own goals. | .91 |
| *Entitlement* | | | | *.72* |
| 5 | 100 | Es mag unfair erscheinen, aber mir steht mehr zu (z.B. Aufmerksamkeit, Privilegien, Belohnungen). | It may seem unfair, but I deserve extra (i.e., attention, privileges, rewards). | .78 |
| 20 | 130 | Ich finde, ich habe einen Anspruch auf eine besondere Behandlung. | I believe I am entitled to special accommodations. | .77 |
| *Lack of Empathy* | | | | *.62* |
| 10 | 86 | Im Allgemeinen schenke ich dem Kummer anderer nicht viel Beachtung. | I don’t generally pay much attention to the woes of others. | .78 |
| 25 | 146 | Das Leiden anderer bekümmert mich nicht. | I don’t get upset with the suffering of others. | .81 |
| *Arrogance* | | | | *1.00* |
| 2 | 12 | Andere sagen, dass ich zu viel angebe, aber alles was ich sage, ist wahr. | Others say I brag too much, but everything I say is true. | .43 |
| 17 | 102 | Ich verschwende meine Zeit nicht damit, mich mit Leuten abzugeben, die meiner nicht würdig sind. | I do not waste my time hanging out with people who are beneath me | .44 |
| *Reactive Anger* | | | | *.45* |
| 13 | 46 | Ich bin manchmal in Rage geraten, wenn ich nicht richtig behandelt wurde. | I have at times gone into a rage when not treated rightly. | .52 |
| 28 | 61 | Es macht mich wirklich wütend, wenn ich nicht bekomme, was mir zusteht. | It really makes me angry when I don’t get what I deserve. | .84 |
| *Distrust* | | | | *.46* |
| 4 | 45 | Wenn jemand etwas Nettes für mich tut, frage ich mich, was er/sie von mir will. | When someone does something nice for me, I wonder what they want from me. | .64 |
| 19 | 135 | Ich denke oft, dass andere mir nicht die ganze Wahrheit erzählen. | I often think that others aren’t telling me the whole truth. | .65 |
| *Thrill Seeking* | | | | *.34* |
| 15 | 89 | Ich würde Verletzungen riskieren, um etwas Aufregendes zu tun. | I would risk injury to do something exciting. | .80 |
| 30 | 134 | Ich mag es, Dinge zu unternehmen, die riskant oder gefährlich sind. | I like doing things that are risky or dangerous. | .88 |
| **Neurotic Narcissism** | | | | |
| *Shame* | | | | *.79* |
| 14 | 107 | Ich schäme mich, wenn andere mich beurteilen. | I feel ashamed when people judge me. | .77 |
| 29 | 122 | Ich fühle mich lächerlich, wenn ich vor anderen einen Fehler mache. | I feel foolish when I make a mistake in front of others. | .81 |
| *Indifference (Reversed)* | | | | *.77* |
| 9 | 33 | Wenn andere mich beurteilen, ist mir das schlicht egal. | When people judge me, I just don’t care. | .73 |
| 24 | 123 | Die Meinungen anderer über mich kümmern mich wenig. | Others’ opinions of me are of little concern to me. | .87 |
| *Need for Admiration* | | | | *1.00* |
| 12 | 4 | Ich habe oft das Gefühl, dass ich von anderen Komplimente brauche, um mir meiner selbst sicher zu sein. | I often feel as if I need compliments from others in order to be sure of myself. | .61 |
| 27 | 124 | Ich wünschte mir, ich würde mich nicht so sehr darum kümmern, was andere von mir denken. | I wish I didn’t care so much about what others think of me. | .82 |

*Note*. LF = Long Form, BF = Brief Form. Factor loadings are based on the hierarchical Confirmatory Factor Analysis Model of the three-factor solution (see manuscript Table 3, model 7).
